# The Alzheimer’s disease risk gene *BIN1* regulates activity-dependent gene expression in human-induced glutamatergic neurons

**DOI:** 10.1038/s41380-024-02502-y

**Published:** 2024-03-22

**Authors:** Orthis Saha, Ana Raquel Melo de Farias, Alexandre Pelletier, Dolores Siedlecki-Wullich, Bruna Soares Landeira, Johanna Gadaut, Arnaud Carrier, Anaïs-Camille Vreulx, Karine Guyot, Yun Shen, Amelie Bonnefond, Philippe Amouyel, Julia TCW, Devrim Kilinc, Claudio Marcos Queiroz, Fabien Delahaye, Jean-Charles Lambert, Marcos R. Costa

**Affiliations:** 1 Univ. Lille, Inserm, CHU Lille, Institut Pasteur de Lille, U1167-RID-AGE facteurs de risque et déterminants moléculaires des maladies liées au vieillissement, DISTALZ, 1 rue du Professeur Calmette, 59019 Lille, France; 2 Brain Institute, Federal University of Rio Grande do Norte, Av. Senador Salgado Filho, 3000, Campus Universitário, Lagoa, Nova, 59078-970 Natal Brazil; 3 Univ. Lille, Inserm, CNRS, CHU Lille, Institut Pasteur de Lille, U1283-UMR 8199 EGID, Pôle Recherche, 1 Place de Verdun, 59045 Lille, Cedex France; 4 Department of Pharmacology, Physiology & Biophysics, Boston University, Chobanian & Avedisian School of Medicine, Boston, MA 02118 USA; 5 Bioinformatics Program, Faculty of Computing & Data Sciences, Boston University, Boston, MA 02115 USA

**Keywords:** Neuroscience, Stem cells, Cell biology

## Abstract

Bridging Integrator 1 (*BIN1*) is the second most important Alzheimer’s disease (AD) risk gene, but its physiological roles in neurons and its contribution to brain pathology remain largely elusive. In this work, we show that *BIN1* plays a critical role in the regulation of calcium homeostasis, electrical activity, and gene expression of glutamatergic neurons. Using single-cell RNA-sequencing on cerebral organoids generated from isogenic *BIN1* wild type (WT), heterozygous (HET) and homozygous knockout (KO) human-induced pluripotent stem cells (hiPSCs), we show that *BIN1* is mainly expressed by oligodendrocytes and glutamatergic neurons, like in the human brain. Both *BIN1* HET and KO cerebral organoids show specific transcriptional alterations, mainly associated with ion transport and synapses in glutamatergic neurons. We then demonstrate that *BIN1* cell-autonomously regulates gene expression in glutamatergic neurons by using a novel protocol to generate pure culture of hiPSC-derived induced neurons (hiNs). Using this system, we also show that *BIN1* plays a key role in the regulation of neuronal calcium transients and electrical activity via its interaction with the L-type voltage-gated calcium channel Cav_1.2_. *BIN1* KO hiNs show reduced activity-dependent internalization and higher Cav_1.2_ expression compared to WT hiNs. Pharmacological blocking of this channel with clinically relevant doses of nifedipine, a calcium channel blocker, partly rescues electrical and gene expression alterations in *BIN1* KO glutamatergic neurons. Further, we show that transcriptional alterations in *BIN1* KO hiNs that affect biological processes related to calcium homeostasis are also present in glutamatergic neurons of the human brain at late stages of AD pathology. Together, these findings suggest that *BIN1*-dependent alterations in neuronal properties could contribute to AD pathophysiology and that treatment with low doses of clinically approved calcium blockers should be considered as an option to slow disease-onset and progression.

## Introduction

The Bridging Integrator 1 (*BIN1*) is the second most important risk locus associated with late-onset Alzheimer’s disease (LOAD), after the Apolipoprotein E (*APOE*) gene [1–4]. In the adult human brain, *BIN1* is mainly expressed by oligodendrocytes, microglial cells and glutamatergic neurons [5–7] and its expression is reduced in AD patients compared to healthy individuals [7, 8]. The consequences of this reduced *BIN1* expression to neuronal and glial cells, as well as the mechanisms by which it contributes to AD pathogenesis remain poorly understood.

*BIN1* has disputably been associated with amyloidopathy and tauopathy, two pathological hallmarks of AD [9]. Reduced *BIN1* expression results in a higher amyloid precursor protein (APP) processing towards the production of amyloid-beta (Aβ) peptides in Neuroblastoma Neuro2a cells [10, 11]. However, we previously showed that *BIN1* knockout (KO) does not increase the concentrations of Aβ peptides in hiPSC-derived neurons (human-induced neurons or hiNs) despite impairing endocytic trafficking [12]. Likewise, reduced *Bin1* expression in the mouse brain does not affect the production of endogenous Aβ peptides [13]. Regarding Tau pathology, decreased expression of the *BIN1* ortholog Amph suppresses Tau-mediated neurotoxicity in Drosophila Melanogaster [14]. In contrast, reduced *Bin1* expression results in higher Tau aggregation and propagation in primary rat hippocampal neurons [15]. In humans, higher concentrations of phosphorylated Tau are observed in the cerebrospinal fluid of patients with AD and are significantly correlated with genetic variants within the *BIN1* locus [16].

More recently, *BIN1* has also been associated with the regulation of synaptic transmission and neuronal electrical activity in animal models [6, 17, 18]. Conditional deletion of *Bin1* in neurons of the adult mice hippocampus leads to altered frequency of mini excitatory post-synaptic currents (mEPSC), likely due to an impaired presynaptic release probability and slower depletion of neurotransmitters [6]. Knockdown of *Bin1* in embryonic rat primary cortical neurons also affects the glutamate AMPA receptor trafficking in the post-synaptic compartment, leading to alterations in the amplitude of mEPSC [18]. Lastly, overexpression of a Bin1-mKate2 fusion protein increases the frequency of spontaneous excitatory postsynaptic currents (sEPSCs) in embryonic rat hippocampal cultures, seemingly by affecting the localization of L-type voltage gated calcium channels (LVGCC) in the membrane through a Tau-dependent interaction [17].

Despite these advances, no consensus has been reached on the roles of *BIN1* in AD pathogenesis and even its physiological functions in human brain cells remain mostly unknown. In this work, we tackled this important question by generating and characterizing human neural cells derived from isogenic *BIN1* wild type (WT), heterozygous (HET) and homozygous knockout (KO) hiPSC lines. We first characterized the transcriptional profile of human neural cells grown in three-dimensional cerebral organoids for more than 6 months and show that reduced BIN1 expression affects mainly glutamatergic neurons. Next, we generated pure *BIN1* WT and KO hiNs cultures and show that BIN1 cell-autonomously regulates electrical activity and gene expression of glutamatergic neurons via the interaction with the LVGCC Cav_1.2_. Pharmacological blockage of this channel with nifedipine partly rescues electrical and gene expression alterations in *BIN1* KO hiNs. Our findings suggest that BIN1 is a key regulator of calcium homeostasis in glutamatergic neurons and that repurposing the use of clinically approved calcium channel blockers could be a promising strategy to treat AD.

## Methods

### hiPSC lines and neural differentiation

Isogenic hiPSCs (ASE 9109, Applied StemCell Inc. CA, USA) modified for *BIN1* in exon 3 were generated by CRISPR/Cas9. Homozygous null mutants for *BIN1* had a 5 bp deletion on one allele and an 8 bp deletion on the other allele. Heterozygous for *BIN1* had a 1 bp insertion on one allele. Whole genome sequencing of the 3 cell lines used in this study confirmed normal ploidy, edits on the *BIN1* locus of HET and KO clones, and absence of other potential CRISPR/Cas9 off-target effects (Supplementary Fig. 1). All hiPSCs, and all subsequent human induced neural progenitor cells (hiNPCs), hiNs, human induced astrocytes (hiAs), and cerebral organoids derived thereof, were maintained in media from Stemcell Technologies. To generate pure neuronal hiNs culture, we expressed Ascl1 in hiNPCs. Maintenance of cell cultures and cerebral organoids are detailed in the supplementary material.

### Electrophysiological recordings and analyses

ASCL1-hiNs were cultured in microfluidic devices bound to multi-electrode arrays (256MEA100/30iR-ITO, Multi-Channel Systems, Germany) and extracellular action potentials were recorded in 5 different cultures for both genotypes at 2, 3, 4 and 6 weeks of differentiation using the MEA2100-256-System (Multi-Channel Systems). For rescue experiments, ASCL1-hiNs were cultured on MEA 96-well plates (CytoView MEA 96, Axion Biosystems, USA) and extracellular action potentials were recorded in 3 independent cultures for either genotype in the presence of 50 nM nifedipine (Tocris Bioscience) or vehicle using the MaestroPro (Axion Biosystems, Inc, USA). Channels containing detected waveforms were processed offline for spike waveform separation and classification using Offline Sorter v3 (Plexon, USA).

### Single-nucleus RNA-sequencing (snRNAseq)

Nuclei isolation and Hash Tag Oligonucleotide (HTO) libraries preparation were performed as previously described [12]. Bioinformatics analyses were performed using Seurat (https://cran.r-project.org/web/packages/Seurat/index.html), Harmony (https://github.com/immunogenomics/harmony), CellID (https://bioconductor.org/packages/release/bioc/html/CelliD.html) and FGSEA (https://bioconductor.org/packages/release/bioc/html/fgsea.html) R packages.

### Statistical analyses

Statistical analyses were performed using GraphPad Prism version 8.0.0 (GraphPad Software, San Diego, California USA, www.graphpad.com) and R 4.2.0 (R Core Team, 2022, https://cran.r-project.org/bin/windows/base/old/4.2.0/). Non-parametric distribution of data was verified using the Kolmogorov-Smirnov test, and dot plots with individual values and median were used to represent these data. For large sample sizes, box plots show 1-99 percentile, outliers and median. Sample sizes, statistical tests and p values are indicated in Figure legends.

## Results

### BIN1 HET and KO cerebral organoids show transcriptional alterations associated with neuronal functional properties

**Fig. 1:**
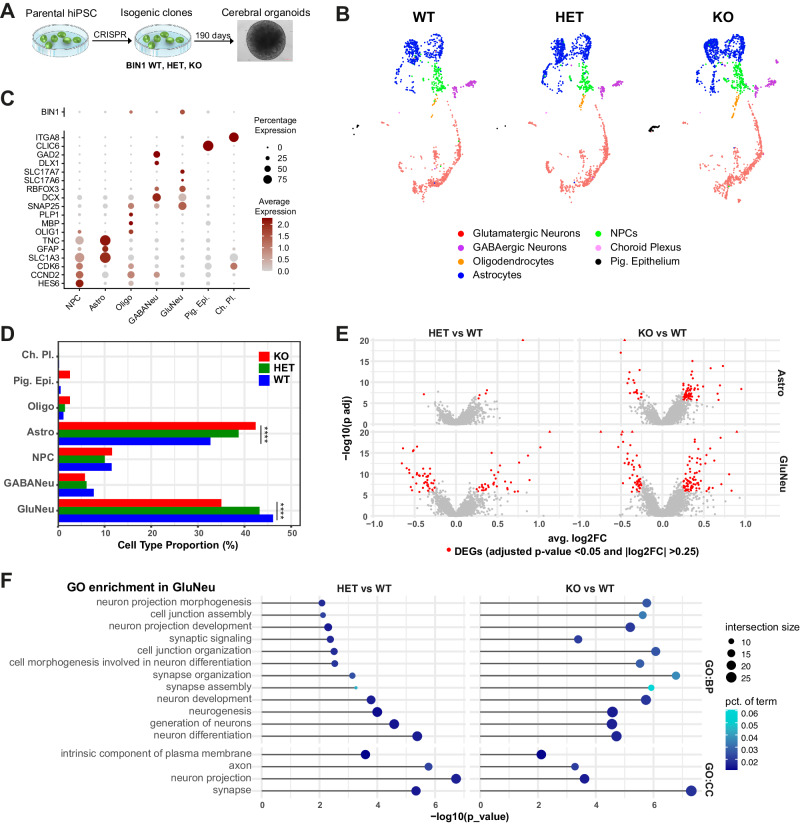
Similar transcriptional changes in glutamatergic neurons from 6.5-month-old *BIN1* HET and KO COs. **A** Scheme of the experimental design. **B** UMAP representation of the different cell subtypes in COs identified using snRNA-seq (*n* = 4 COs per genotype). **C** Dot plot representing the expression for *BIN1* and key markers used to annotate cell subtypes. **D** Proportion of cell subpopulations in the three genotypes (****p < 0.0001; Chi-squared test). **E** Volcano plots representing DEGs comparing HET vs WT or KO vs WT in astrocytes and glutamatergic neurons. DEGs with adjusted *p* value < 0.05 and |log2FC | >0.25 are shown in red. **F** Functional enrichment analysis of DEGs commonly identified in HET and KO glutamatergic neurons. GO gene ontology, BP biological processes, CC cellular components.

Cerebral organoids (COs) faithfully recapitulate fundamental aspects of the three-dimensional organization of the human brain, including the molecular specification of different neural cell types/subtypes and the generation of complex electrical activity patterns [19, 20]. To investigate the potential role of *BIN1* in human neural cells, we generated and characterized COs using isogenic *BIN1* wild type (WT), heterozygous (HET) and KO hiPSCs (Fig. 1A, Supplementary Fig. 1, Supplementary Table 1). After 6.5 months of culture, COs were composed of all the major neural cell types identified by the expression of MAP2, GFAP and NESTIN and we did not observe any gross difference in size or morphology of COs among the three genotypes (Supplementary Fig. 2A). Western blot analyses confirmed the reduction and absence of BIN1 protein in *BIN1* HET and KO COs, respectively (Supplementary Fig. 2B). We then employed single-nucleus RNA sequencing (snRNA-seq) to further characterize individual cell types/subtypes and investigate possible gene expression alterations associated with reduced *BIN1* expression. COs (*n* = 4 from each genotype) were divided into two halves that were independently processed for western blotting or snRNA-seq. We observed similar expression of general neuronal and glial proteins in these COs (Supplementary Fig. 2C), suggesting a low degree of heterogeneity in these samples. Nevertheless, to further reduce potential batch effects, we pooled COs into a single multiplexed library using Cell Hashing [21]. After sequencing, quality control and demultiplexing, we recovered 4398 singlets that could be grouped into 7 major cell clusters based on the expression of cell type markers *SLC1A3 (GLAST), GFAP* and *TNC* (astrocyte); *SNAP25, DCX and MAPT* (pan-neuronal); *SLC17A7* and *SLC17A6* (glutamatergic neurons); *DLX1, GAD1* and *GAD2* (GABAergic neurons); *HES6, CCND2* and *CDK6* (NPCs); *ITGA8* (choroid plexus); and *CLIC6* (pigmented epithelium) (Fig. 1B, C). *BIN1* expression in COs was mainly detected in glutamatergic neurons and oligodendrocytes (Fig. 1C), similar to the profile described for the human brain [7]—except for brain microglial cells that are not present in COs. We also observed a reduction and enlargement, respectively, in the proportions of glutamatergic neurons and astrocytes in *BIN1* KO compared to WT (Fig. 1D; ****p < 0.0001; Chi-square test).

To identify possible differentially expressed genes (DEGs) in *BIN1* KO or HET compared to WT cells, we performed a Wilcoxon test for each major cell type/subtype identified in COs. Consistent with the predominant expression of *BIN1* in glutamatergic neurons (Fig. 1D), we identified a high number of DEGs in this cell type both in *BIN1* HET (76 genes) and KO (124 genes) compared to WT genotype (Figure1E; Supplementary Fig. 3A). Furthermore, we found that *BIN1* HET and KO glutamatergic neurons shared a significant proportion of DEGs and that correlation between gene expression changes in these two independent clones was highly significant (Supplementary Fig. 3B–D). In astrocytes, we also detected 75 DEGs in *BIN1* KO, but only 6 DEGs in HET compared to WT (Fig. 1E; Supplementary Fig. 3A). For all other cell types, we observed a maximum of 1–4 DEGs in the comparison between *BIN1* KO vs WT or HET vs WT (Supplementary Table 2). These observations suggest that both *BIN1* null (KO) and partial deletion (HET) affect similar biological processes in glutamatergic neurons in a dose-dependent manner. Accordingly, similar GO terms were enriched for DEGs identified in *BIN1* KO or HET glutamatergic neurons, including several terms associated with synaptic transmission (Fig. 1F). For *BIN1* KO glutamatergic neurons, we also identified GO terms associated with ion channel complex and calcium ion binding (Supplementary Fig. 3E; Supplementary Table 3), further suggesting that reduced *BIN1* expression leads to specific transcriptional changes associated with functional properties of this neuronal subtype. DEGs identified in astrocytes of *BIN1* KO compared to WT COs significantly enriched for terms associated with nervous system development and cell migration (Supplementary Table 3).

### Altered expression of activity-related genes in BIN1 KO and HET COs

**Fig. 2:**
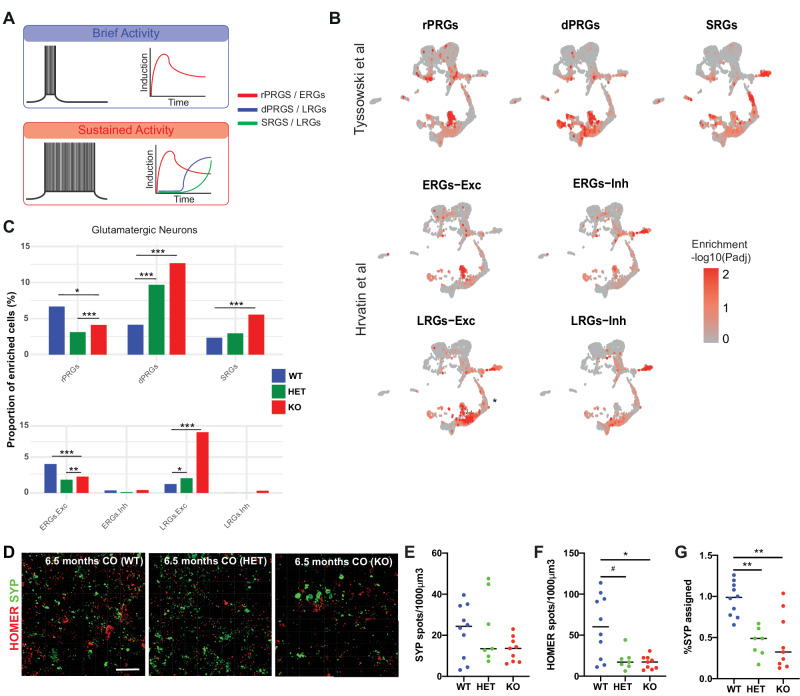
Altered expression of activity-related genes and reduced synaptic numbers in *BIN1* HET and KO glutamatergic neurons. **A** Scheme indicating the different sets of ARGs regulated by brief and sustained patterns of electrical activity [23, 24]. rPRGs: rapid primary response genes; dPRGs: delayed primary response genes; SRGs: secondary response genes; ERGs early response genes, LRGs late response genes, Exc glutamatergic neurons, Inh GABAergic neurons. **B** Feature plots showing the enrichment score of single cells for ARG signatures. Enrichment scores correspond to the –log10(p_adj_) of the Cell-ID-based enrichment test. **C** Proportions of glutamatergic neurons enriched for the different ARG signatures according to genotype (*p < 0.05; **p < 0.01; ***p < 0.001; Chi-squared test). **D** Immunohistochemistry for HOMER1 (red), SYP (green) in 6.5-month-old *BIN1* WT, HET and KO COs. **E**–**G** Quantifications of the SYP and HOMER1 spot density, and the percentage of SYP assigned by HOMER1 spots in *BIN1* WT, HET and KO COs (^#^*p* < 0.1; *p < 0.05; **p < 0.01; Dunn’s multiple comparison test; *n* = 3 COs per genotype).

Neuronal firing patterns (such as tonic and burst firing) play a key role in the transcriptional regulation of a particular set of genes designated activity-related genes (ARGs) [22]. While neurons stimulated with brief patterns of electrical activity transcribe rapid primary response genes (rPRGs) or early response genes (ERGs), those stimulated with sustained patterns of electrical activity express delayed primary response genes (dPRGs), secondary response genes (SRGs) or late response genes (LRGs) (Fig. 2A) [23, 24]. Using Cell-ID [25], we quantified the enrichment for ARGs signatures (Supplementary Table 4) in COs at single-cell resolution as an indirect readout of neuronal electrical activity patterns in this model. We first confirmed that ARG signatures were predominantly enriched in neurons (Fig. 2B). Then, we quantified the proportion of glutamatergic or GABAergic neurons significantly enriched for such specific responses in gene signatures (*p*_adj_ < 0.05; hypergeometric test). We observed a significantly higher proportion of glutamatergic neurons enriched for dPRGs and LRGs both in *BIN1* HET and KO, as well as SRGs in *BIN1* KO compared to WT glutamatergic neurons, whereas the proportion of glutamatergic neurons enriched for rPRGs and ERGs was reduced in *BIN1* HET and KO (Fig. 2C). In sharp contrast, the only difference observed in GABAergic neurons was a reduction in the proportion of cells enriched for SRGs (Supplementary Fig. 4). These results suggest that reduced *BIN1* expression in glutamatergic neurons triggers neuronal firing patterns towards sustained activity leading to a higher expression of late-response ARGs.

### Lower numbers of synaptic puncta in BIN1 HET and KO COs compared to WT

Transcriptional alterations in *BIN1* HET and KO glutamatergic neurons are also suggestive of synaptic dysfunction, which is an early hallmark of AD pathology [26]. We thus sought to determine whether reduced *BIN1* expression could affect synaptic connectivity in COs. Using immunohistochemistry to detect the expression of the pre-synaptic protein Synaptohysin-1 (SYP) and post-synaptic protein HOMER1, we were able to quantify the frequency of putative synaptic contacts (% SYP assigned) in COs (see methods). We observed a significant reduction in the percentage of SYP assigned both in *BIN1* HET and KO compared to WT, mainly due to a reduction in the number of post-synaptic spots expressing HOMER1 (Fig. 2D–G).

Next, we investigated whether APP processing and Tau phosphorylation, which have been previously associated with *BIN1* and are known to modulate neuronal electrical activity [10, 15, 27], could also be altered in *BIN1* HET and KO COs. To this end, we measured the intracellular levels of full-length APP and APP β-CTF (as a readout of amyloidogenic APP processing), total and phosphorylated TAU proteins by western blotting. Besides a trend for reduced TAU expression, likely explained by the reduced proportion of neurons (Fig. 1), we did not detect any significant differences in the intracellular levels of APP, APP β-CTF, TAU or phospho-TAU (Ser202, Thr205) in *BIN1* HET and KO compared to WT COs (Supplementary Fig. 5). Altogether, these results suggest that reduced *BIN1* expression could alter neuronal functional properties without significantly affecting APP or Tau metabolism in COs.

### Cell-autonomous role of BIN1 in the regulation of neuronal gene expression

**Fig. 3:**
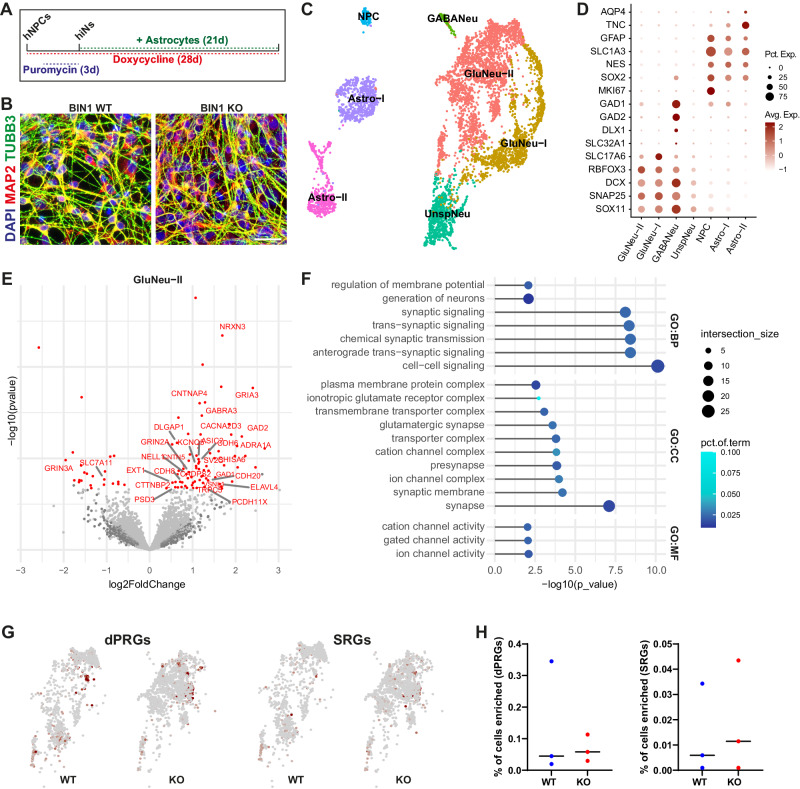
Neuronal-specific *BIN1* KO cell-autonomously elicit transcriptional changes. **A** Scheme of the Ascl1-induced hiNs experiments. **B** Images showing *BIN1* WT and KO hiNs 7 days after the beginning of doxycycline treatment immunolabeled for neuronal markers MAP2 and TUBB3 and astrocyte marker GFAP and stained with DAPI. Scale bar: 50 μm. **C** UMAP representation of the different cell subtypes identified in ASCL1-hiNs cultures using snRNA-seq (*n* = 3 independent culture batches). **D** Dot plot representing expression of key markers used to annotate cell subtypes. **E** Volcano plot representing DEGs comparing *BIN1* KO vs WT glutamatergic neurons (GluNeu-II). Differential expression analysis was performed using DEseq2 on the sample level gene expression matrix, including the experiment batch as covariates. DEGs with adjusted *p* value < 0.05 and |log2FC | >0.25 are shown in red. Gene labels are shown for calcium- and synapse-related genes. **F** Functional enrichment analysis of DEGs identified in glutamatergic neurons. Bar plots representing the top 15 the enriched GO terms in each category at adjusted *p* value < 0.01. **G** UMAP representing GluNeu-II enrichment for dPRGs and SRGs (Tyssowski et al., 2018). Red points indicate enrichment with adjusted *p* value < 0.05, hypergeometric test using CellID. **H** Dot plots showing percentage of GluNeu-II cells enriched for dPRGs and SRGsby genotype in 3 independent cultures.

Our results in COs suggest that reduced *BIN1* expression affects mainly glutamatergic neurons. However, at least in *BIN1* KO COs, we cannot completely rule out an effect of *BIN1* deletion in astrocytes that could indirectly impact glutamatergic neurons. Therefore, to unambiguously probe the cell-autonomous effect of *BIN1* deletion on the electrical activity and gene expression of human glutamatergic neurons, we generated *BIN1* WT or KO pure neuronal cultures by direct lineage-reprogramming of hiPSC-derived neural progenitor cells NPCs (hiNPCs) using doxycycline-inducible expression of ASCL1 (Fig. 3A; see Supplementary Methods). After validation of the efficient lineage-reprogramming of hiNPCs into highly pure neurons (hereafter ASCL1-hiNs) (Fig. 3B), we added exogenous human cerebral cortex astrocytes to trophically support functional neuronal maturation and synaptic connectivity [28]. Using snRNA-seq after 4 weeks of differentiation we identified 5583 cells (*n* = 3 independent culture batches) clustered into two main glutamatergic neuron (GluNeu-I and II), one GABAergic neuron (GABANeu), one immature/unspecified neuron (UnspNeu), two astrocytes (Astro-I and II) and one proliferative NPC groups (Fig. 3C, D). Sample-level differential gene expression analysis using DESeq2 [29], revealed 99 DEGs ( | log2FC | >0.25 and FDR < 0.05) in *BIN1* KO GluNeu-II compared to WT, but only two in GluNeu-I and one in immature neurons (Fig. 3E; Supplementary Table 5, Supplementary Fig. 6). As observed in COs (Fig. 1H–I), GO term enrichment analysis revealed a significant enrichment for terms associated with synaptic transmission, ion channel activity and calcium signaling pathways (Fig. 3F; Supplementary Fig. 6; Supplementary Table 6). The percentage of GluNeu-II enriched for late-response ARGs was slightly greater in *BIN1* KO compared to WT, but without statistical differences (Fig. 3G, H). Exogenously added human astrocytes co-cultured with *BIN1* WT and KO hiNs also showed a low number of DEGs (11 in Astro-I; Supplementary Table 5), likely reflecting an astrocyte reaction to primary changes in hiNs in response to *BIN1* deletion.

### *BIN1* KO leads to alteration in the electrical activity pattern of ASCL1-hiNs

**Fig. 4:**
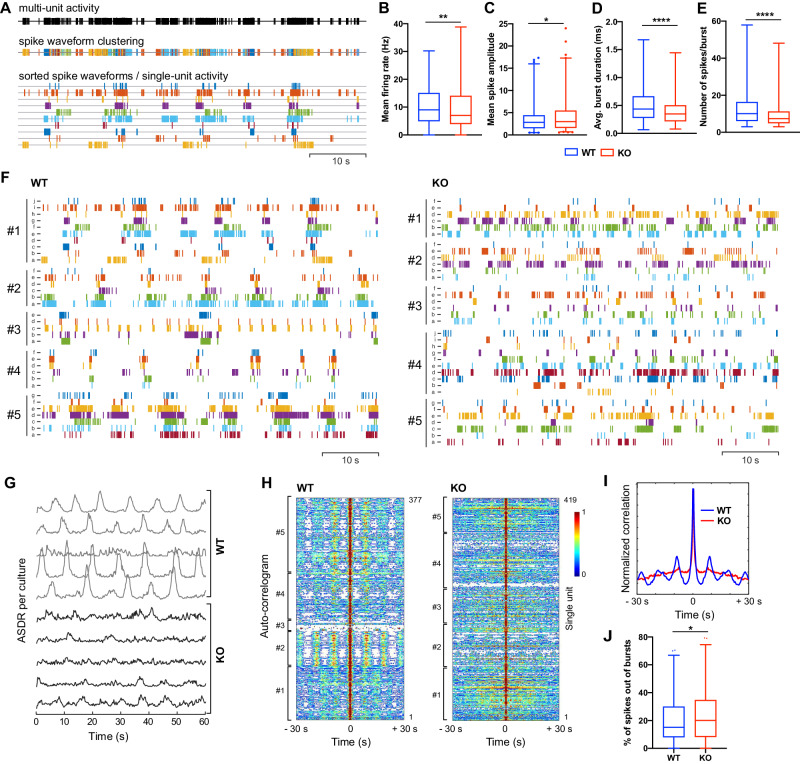
Disorganization of neuronal activity in *BIN1* KO ASCL1-hiNs. **A** Raster plots showing the decomposition of multi-unity activity (MUA, black lines) into single-unit activity (SUA, colored lines) using spike waveform clustering. Quantification of single-neuron firing rate (**B**; **p = 0.0034), spike amplitude (**C**; *p = 0.0106), burst duration (**D**; ****p < 0.0001) and number of spikes per burst (**E**; ****p < 0.0001) at 4 weeks (Mann–Whitney test; *n* = 5 independent experiments; WT: 376 neurons; KO: 416 neurons). **F** Raster plots showing SUA recorded from 5 different electrodes of *BIN1* WT (left) or KO (right) ASCL1-hiNs cultures after 4 weeks of differentiation. **G** Array-wide spike detection rate (ASDR) plots based on SUA recorded in *BIN1* WT and KO ASCL1-hiNs cultures. Each line represents one independent culture batch. Normalized autocorrelogram heatmap (**H**, each line refers to one SUA) and averaged correlation (**I**) for all SUAs recorded in 5 independent *BIN1* WT and KO ASCL1-hiNs cultures. **J** Percentage of spikes outside of bursts (*p = 0.0417, Mann–Whitney test).

The transcriptional changes observed in our 2D and 3D models could suggest a role of *BIN1* in regulating functional properties of glutamatergic neurons. To directly address this possibility, we used multi-electrode arrays (MEA) to record and quantify multi-unit activity (MUA) in ASCL1-hiNs. As previously described in spontaneously differentiated hiPSC-derived neuronal cultures [30], ASCL1-hiNs cells exhibited a diverse range of spontaneous activity patterns, including regular discharges, population bursts and period activity (Supplementary Fig. 7A). In this respect, we found a conspicuous change in the temporal organization of MUA after *BIN1* deletion (Supplementary Fig. 7A), mainly characterized by a greater number of spike bursts at 4 weeks (Supplementary Fig. 7D). These alterations may result from changes at the single cell or the population level (different number of neurons contributing to each electrode, for example). To disentangle these possibilities, we used waveform-based spike sorting to examine the functional consequences of *BIN1* deletion at the single neuronal level (Fig. 4A). We identified a similar number of single units per recording electrode between genotypes (WT: 4.92 ± 2.34; KO: 5.27 ± 2.45), indicating that *BIN1* deletion does not affect the density of active neurons within culture. However, we observed reduced single-unit activity (SUA) frequency (Fig. 4B) and higher SUA amplitude (Fig. 4C) in *BIN1* KO compared to WT ASCL1-hiNs. Interestingly, we could not detect significant changes in the number of bursts per neuron (WT: 11.01 ± 6.71; KO: 10.36 ± 8.59), although both the burst duration and the number of spikes within a burst were significantly lower in *BIN1* KO compared to WT ASCL1-hiNs (Fig. 4D, E). We also observed a prominent temporal disorganization of *BIN1* KO hiNs activity by computing the array-wide spike detection rate (ASDR, Fig. 4G), which reveals the strength of the synchronized population activity, and the autocorrelograms of SUAs (Fig. 4H, I), which allows the apprehension of synchronized periodicity. These analyses revealed that most spikes of *BIN1* WT neurons were organized in bursts occurring at periodic intervals of about 8-10 s, whereas the spikes of *BIN1* KO neurons were randomly distributed, leading to a higher percentage of spikes occurring outside of bursts compared to WT neurons (Fig. 4J).

### Altered electrical activity of *BIN1* KO ASCL1-hiNs is associated with normal synapse numbers and altered TAU phosphorylation

**Fig. 5:**
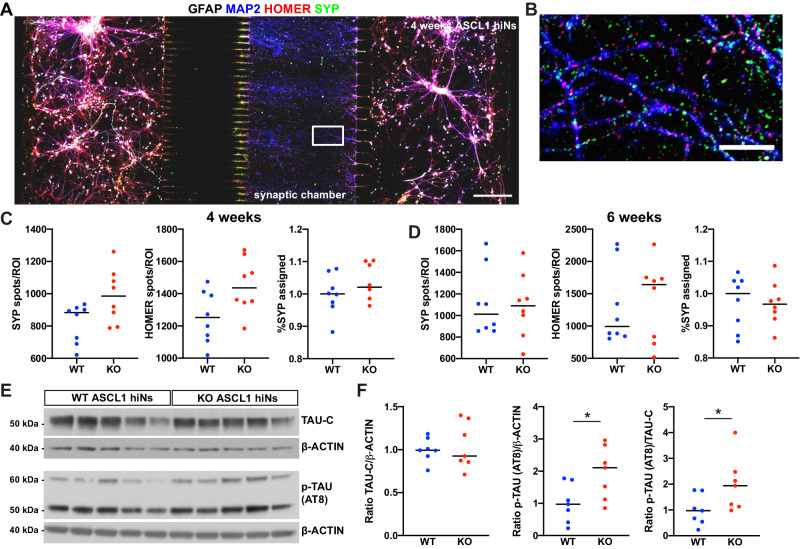
Similar synaptic density and higher TAU phosphorylation in *BIN1* KO compared to WT ASCL1-hiNs. **A**, **B** Immunocytochemistry using the astrocyte marker GFAP, neuronal marker MAP2, pre-synaptic marker SYP and post-synaptic marker HOMER1 in *BIN1* WT ASCL1-hiNs after 4 weeks of differentiation in a three-chamber microfluidic device. Scale bar = 200 μm. Rectangular box in (**A**) is magnified in (**B**), allowing the identification of putative synaptic contacts. Fraction of SYP spots assigned by HOMER1 spots in MAP2 processes at 4 (**C**) and 6 weeks (**D**) ASCL1-hiNs cultures (*n* = 8 microfluidic devices per genotype from 2 independent differentiations). **E** Western blot for total TAU protein C-terminal (TAU-C), phosphorylated (p)-TAU at Ser202, Thr205 (AT8) and β-ACTIN in 4-week-old ASCL1-hiNs cultures. **F** Quantification of TAU-C/β-ACTIN, p-TAU/ β-ACTIN and p-TAU/TAU-C levels in *BIN1* KO ASCL1-hiNs normalized to WT (*p = 0.0379 for p-TAU/ β-ACTIN and *p* = 0.0262 for p-TAU/TAU-C; Mann–Whitney test; *n* = 7 independent culture batches). Uncropped plots are shown in Supplementary Fig. 12.

The changes in neural network activity observed in *BIN1* KO hiNs could be explained by, among other things, a reduced synaptic connectivity, as observed in our long-term COs cultures (Fig. 2). To test this possibility, we first quantified the number of synaptic contacts in *BIN1* WT and KO ASCL1-hiNs cultures. In contrast with COs, we did not detect any significant differences in the number of putative synaptic contacts (% SYP assigned) in *BIN1* KO compared to WT ASCL1-hiNs, neither after 4 nor 6 weeks of differentiation (Fig. 5A–D). Next, we quantified the number and activity of glutamatergic synapses by using real-time imaging of ASCL1-hiNs expressing the glutamate sensor iGLUSnFr [31]. In accordance with our observations based on immunocytochemistry, we did not detect differences neither in the number of glutamatergic synapses (active spots) nor in the frequency of events (change in fluorescence levels in active spots) in *BIN1* KO compared to WT ASCL1-hiNs (Supplementary Fig. 8; Supplementary Movies 1 and 2), indicating that changes in neuronal activity observed in our cultures are not related to changes in synaptic transmission.

Taking advantage of this culture system comprising enriched neuronal populations, we also sought to confirm whether *BIN1* deletion could be associated with changes in APP processing or Tau phosphorylation. To this end, we measured the extracellular levels of amyloid-beta (Aβ) peptides, as well as the intracellular levels of full-length APP and APP β-CTF, total and phosphorylated TAU proteins in ASCL1-hiNs cultures. Like COs, we did not detect any significant differences neither in the extracellular levels of Aβ1-x or Aβ1-42, nor in the intracellular levels of APP or APP β-CTF in *BIN1* KO compared to WT ASCL1-hiNs (Supplementary Fig. 9). However, in contrast with our observations in COs, we observed significantly higher levels of phospho-TAU (Ser202, Thr205) relative to β-ACTIN and total TAU in in *BIN1* KO compared to WT ASCL1-hiNs (Fig. 5E, F). Together, these observations may suggest that *BIN1* deletion primarily impairs neuronal intrinsic properties regulating electrical activity and Tau phosphorylation prior to detectable changes in synaptic numbers (observed only in long-term cultures – Fig. 2) and independently of alterations in APP processing.

### BIN1 regulates neuronal Ca^2+^ dynamics through Cav_1.2_

**Fig. 6:**
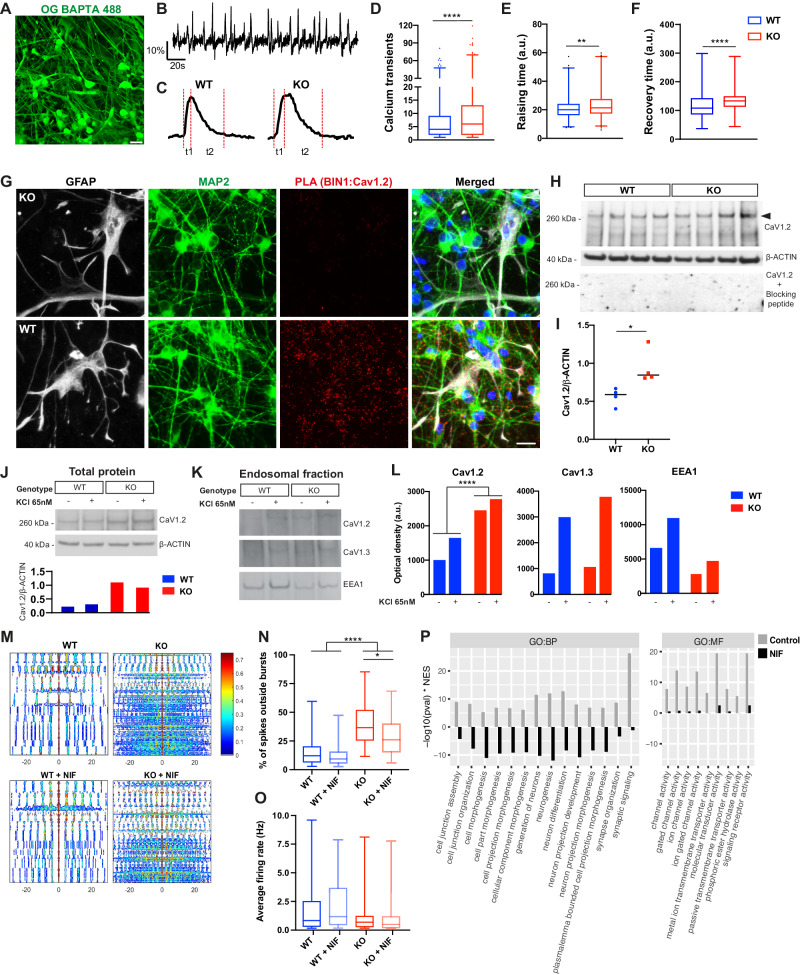
Altered frequency of calcium transients in *BIN1* KO ASCL1-hiNs. **A** Snapshot of a 4-week-old ASCL1-hiNs culture labeled with Oregon green BAPTA. **B** Representative plot of fluorescence changes over time in 1000 frames. **C** Representative traces showing the fluorescence changes in *BIN1* WT and KO ASCL1-hiNs. Red dashed lines indicate the time to reach the fluorescence maximal intensity (raising time - t1) and to return to baseline (recovery time - t2). **D** Quantification of calcium transients in *BIN1* WT and KO ASCL1-hiNs (****p < 0.0001; Mann–Whitney test; *n* = 3 independent cultures for each genotype; number of active cells per condition: 754 (WT), 1006 (KO)). **E**, **F** Quantification of rising time (t1) and recovery time (t2) for calcium transients (**p = 0.0022; ****p < 0.0001; Mann–Whitney test). **G** Images showing PLA spots using anti-BIN1 and anti-Cav_1.2_ antibodies in 4-week-old *BIN1* WT and KO hiNs. Cells were also immunolabeled for the neuronal marker MAP2 (green), the astrocyte marker GFAP (white), and stained with DAPI (blue). **H** Western blot for Cav_1.2_ (without and with blocking peptide) and β-ACTIN in 4-week-old ASCL1-hiNs cultures. **I** Quantification of Cav_1.2_/β-ACTIN levels in *BIN1* WT and KO ASCL1-hiNs cultures (*p = 0.0286; Mann–Whitney test; *n* = 4 independent culture batches). **J** Western blot for Cav_1.2_ and β-ACTIN in the total protein extracts from 4-week-old ASCL1-hiNs treated with KCl (+) or vehicle (–). Plot shows the quantification of Cav_1.2_ normalized by β-ACTIN. **K** Western blot for Cav_1.2_, Cav_1.3_ and EEA1 in the endosomal protein extracts from 4-week-old ASCL1-hiNs treated with KCl (+) or vehicle (–). **L** Plot shows the optical density of these proteins (****p < 0.0001; Chi-square test; *n* = 6 independent cultures for each genotype/treatment pooled). Uncropped plots are shown in Supplementary Figs. 13, 14. **M** Auto-correlograms of 4-week-old *BIN1* WT and KO hiNs treated or not with 50 nM Nifedipine (NIF) for 2 weeks. **N** Percentage of spikes outside of bursts (WT or WT + NIF vs KO or KO + NIF: ****p_adj_ < 0.0001; KO vs KO + NIF: *p_adj_ = 0.0124; Dunn’s multiple comparison test; *n* = 3 independent culture batches). **O** Average firing rates. **P** Comparison of the enrichment for GO terms in genes upregulated in KO vs WT and KO + nifedipine (NIF) vs WT GluNeu-II using fast gene set enrichment analysis (FGSEA). The FGSEA results are shown for the top15 GO terms with reduced enrichment in *BIN1* KO ASCL1-hiNs treated with nifedipine (NIF) or vehicle (Control) compared to WT cells.

In neurons, electrical activity is always accompanied by an influx of Ca^2+^ ions, which play a fundamental role in the regulation of neuronal firing and activity-dependent gene transcription [32]. We therefore postulated that reduced *BIN1* expression in human glutamatergic neurons could primarily affect Ca^2+^ dynamics, as previously suggested for cardiomyocytes [33]. To directly test this possibility, we first studied Ca^2+^ dynamics in *BIN1* WT and KO ASCL1-hiNs using real-time calcium imaging experiments. We observed spontaneous, synchronous calcium transients among adjacent cells both in *BIN1* WT and KO ASCL1-hiNs cultures (Supplementary Movies 3 and 4). By quantifying calcium spike transients (>2 standard deviations above the noise level), we showed a significantly higher frequency of Ca^2+^ transients in *BIN1* KO compared to WT ASCL1-hiNs (Fig. 6A, B, D). Moreover, the dynamics of individual Ca^2+^ transients in *BIN1* KO were qualitatively different from WT ASCL1-hiNs (Fig. 6C). These differences could be quantitatively measured by a longer time to reach the maximum intracellular Ca^2+^ levels and to recover baseline levels (Fig. 6E, F).

In human heart failure, *BIN1* expression is reduced, leading to an impairment in Cav_1.2_ trafficking, calcium transients, and contractility [33]. Thus, we sought to determine if BIN1 could also interact and regulate LVGCC expression in human neurons. To this end, we performed proximity ligation assay (PLA) to probe a possible interaction between BIN1 and Cav_1.2_ or Cav_1.3_, the two LVGCCs expressed in ASCL1-hiNs (Supplementary Fig. 10). We observed a widespread BIN1-Cav_1.2_ PLA signal (Fig. 6G) and, to a lesser extent, a BIN1-Cav_1.3_ PLA signal in neurons (Supplementary Fig. 10). Next, we quantified neuronal LVGCC protein by western blotting and observed higher total Cav_1.2_ expression in *BIN1* KO compared to WT ASCL1-hiNs (Fig. 6H–I). Protein expression of neither Cav_1.3_, nor the members of the Cav_2_ family (Cav_2.1_, Cav_2.2_ and Cav_2.3_) were altered in the same cultures (Supplementary Fig. 10), suggesting a specific regulation of Cav_1.2_ expression by BIN1.

Notably, LVGCCs are key regulators of neuronal firing [30] and activity-dependent internalization of these channels is a key mechanism in firing homeostasis [34]. We thus set out to investigate whether *BIN1* deletion could impair this mechanism in human neurons. We stimulated ASCL1-hiNs with KCl 65 nM for 30 min to induce neuronal depolarization and collected total and endosomal proteins for analysis. We confirmed a higher global level of Cav_1.2_ in *BIN1* KO compared to WT ASCL1-hiNs that was independent of KCl treatment (Fig. 6J). We also confirmed that the treatment with KCl 65 nM for 30 min was sufficient to increase the endosomal fraction expressing the early endosome antigen 1 (EEA1) and the internalization of Cav_1.3_ in both *BIN1* WT and KO ASCL1-hiNs (Fig. 6K–L). However, while Cav_1.2_ expression in the endosomal fraction was 50% higher after KCl treatment in *BIN1* WT, this rise was only 10% in *BIN1* KO ASCL1-hiNs (Fig. 6K–L). These results indicate that BIN1 interacts and regulates the activity-dependent internalization of Cav_1.2_ in human neurons.

### Treatment with the calcium channel blocker nifedipine partly rescues electrical and gene expression alterations in BIN1 KO ASCL1-hiNs

To investigate whether the network dysfunctions observed in *BIN1* KO ASCL1-hiNs could be related to the altered expression of Cav_1.2_ in these cells, we treated cultures with a physiologically relevant concentration (50 nM) of the Cav_1.2_ blocker nifedipine [35] for 2 weeks and recorded neuronal activity using MEA electrophysiology. We observed a partial recovery of the oscillatory pattern of neuronal electrical activity observed in WT cells (Fig. 6M). Interestingly, the percentage of spikes outside bursts was not affected by nifedipine treatment in *BIN1* WT but was significantly lower in *BIN1* KO ASCL1-hiNs (Fig. 6N), indicating a partial recovery of burst organization. To note, no difference in firing rates was observed whatever the models and conditions (Fig. 6O). After 2 weeks of nifedipine treatment (4 weeks of differentiation), we also performed snRNA-seq experiments and recovered a total of 1537 cells (*n* = 2 independent culture batches), which were mapped into the 7 clusters described earlier (Fig. 3; Supplementary Fig. 11). Using the Wilcoxon test, we found that nifedipine treatment down-regulated several genes in *BIN1* KO ASCL1-hiNs, especially in the GluNeu-II population (Supplementary Table 7). The downregulated genes after nifedipine treatment were enriched for LRGs of excitatory neurons, suggesting rescue of the altered activity-related gene expression (Supplementary Fig. 11D). Furthermore, several GO terms associated with ion channel activity and synapse transmission, which are found enriched in *BIN1* KO vs WT GluNeu-II population, show consistently reduced enrichment in nifedipine treated KO cells (Fig. 6P; Supplementary Table 8). Altogether, these data support the view that BIN1 contributes to the regulation of electrical activity and gene expression through the regulation of Cav_1.2_ expression/localization in human neurons.

### Molecular alterations in *BIN1* KO organoids and ASCL1-hiNs are also present in glutamatergic neurons of AD patients

**Fig. 7:**
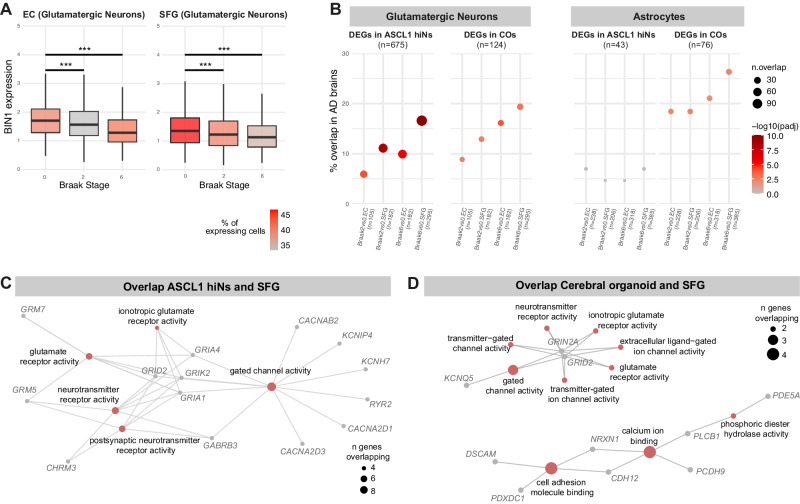
Similar molecular alterations in *BIN1* KO hiNs and glutamatergic neurons of the AD brain. **A** Box plot representing *BIN1* mRNA in expression through different Braak stages in the entorhinal cortex (EC) and superior frontal gyrus (SFG) of AD patients at different Braak stages (***p_adj_ < 0.001; Wilcoxon test). **B** Dot plot representing the overlap between DEGs identified in glutamatergic neurons of the AD brain and *BIN1* KO ASCL1-hiN cultures (left) or *BIN1* KO COs (right). **C**, **D** Network representation of enriched GO terms in overlapping DEGs between AD brains and glutamatergic neurons in culture.

We finally sought to evaluate whether molecular alterations in our neural models may recapitulate some of those observed in the brains of AD cases. For this purpose, we used a publicly available snRNA-seq dataset generated from the entorhinal cortex (EC) and superior frontal gyrus (SFG) of AD patients at different Braak stages [36]. We first observed a progressive and significant decrease in *BIN1* mRNA levels in glutamatergic neurons of both brain regions (Fig. 7A), suggesting that reduced *BIN1* expression in this cell type may be a common feature occurring in the AD pathology progression. We then compared DEGs identified in *BIN1* KO glutamatergic neurons (either from COs or ASCL1-hiNs) with those identified in the same cell subtype of AD brains (Supplementary Table 9). Remarkably, DEGs identified in *BIN1* KO glutamatergic neurons (either from COs or ASCL1-hiNs) showed a statistically significant overlap with DEGs detected in this cell population of AD brains at different Braak stages (Fig. 7B). In astrocytes, however, a similar significant overlap could only be observed between COs and AD brains. GO analysis based on DEG overlap between *BIN1* KO ASCL1-hiNs and AD brain glutamatergic neurons indicated significant enrichment for pathways associated with glutamate receptor activity and gated channel activity (Fig. 7C; Supplementary Table 8). Similarly, DEG overlap between *BIN1* KO COs and AD brain glutamatergic neurons was significantly enriched for genes associated with glutamate receptor activity, gated channel activity and calcium ion binding (Fig. 7D; Supplementary Table 8). No significant enrichment was observed for DEG overlap between *BIN1* KO COs and AD brain astrocytes (data not shown). Therefore, gene expression alterations in *BIN1* KO hiNs and glutamatergic neurons of AD patients overlap and converge to biological processes associated with calcium homeostasis.

## Discussion

In this work, we show that the AD genetic risk factor *BIN1*, plays a critical role in the regulation of neuronal firing homeostasis and gene expression in glutamatergic neurons. Complete deletion of *BIN1* is sufficient to alter the expression of the LVGCC Cav_1.2_, leading to altered calcium homeostasis and neural network dysfunctions in human neurons in vitro. These functional changes are correlated with changes in the expression of genes involved in synaptic transmission and ion transport across the membrane, as well as elevated Tau phosphorylation. In long-term neuronal cultures using COs, we show that reduced *BIN1* expression is associated with fewer synapses and specific gene expression alterations in glutamatergic neurons associated with activity-dependent transcription. Notably, reduced *BIN1* expression in human-induced glutamatergic neurons is sufficient to elicit gene expression alterations that are also present in AD and converge to biological processes related with calcium homeostasis and synaptic transmission. Together, our findings support the view that altered *BIN1* expression in glutamatergic neurons may contribute to AD pathophysiology by dysregulating neuronal firing homeostasis via LVGCCs.

We also show using our new protocol to generate pure ASCL1-hiNs cultures that deletion of *BIN1* expression only in neurons is sufficient to increase the phosphorylation of TAU but does not increase amyloidogenic APP processing, as previously shown in other hiPSC-derived neuronal cultures [12]. In addition, in 6.5 months-old cerebral organoids, we observe a 40% higher ratio of p-Tau/Tau in *BIN1* KO compared to WT genotype. These results further support the notion that *BIN1* expression, at least in neurons, contributes to regulate Tau phosphorylation/propagation [15, 37] and are in agreement with several lines of evidence showing a significant association between the *BIN1* locus with elevated total Tau/p-Tau in the brain and cerebrospinal fluid of AD patients [16, 38]. It would be interesting to investigate in the future whether the altered electrical activity observed in hiNs expressing reduced levels of *BIN1* are a cause or a consequence of the higher levels of phosphorylated Tau in these cells [17, 39].

Neuronal network dysfunctions are observed in AD patients at early stages of the disease and precede or coincide with cognitive decline [40–42]. Under physiological conditions, neuronal networks can maintain optimal output through regulation of synaptic plasticity and firing rate [43]. Our results suggest that normal levels of *BIN1* expression in glutamatergic neurons are fundamental in regulating neuronal firing rate homeostasis. Accordingly, *BIN1* KO in hiNs is sufficient to dysregulate network oscillations even without impacting the number of functional synaptic contacts, suggesting that the desynchronization observed in *BIN1* KO hiNs circuits are a consequence of disordered homeostatic controls of neuronal activity. In long-term hiNs cultures (COs), glutamatergic neurons show both gene expression alterations indicative of altered electrical activity and reduced synaptic densities, which could indicate a synaptic down-scaling in response to earlier augmented electrical activity [44].

One key mechanism controlling neuronal spiking activity is the regulation of Ca^2+^ homeostasis [30, 32, 45]. Boosted neuronal electrical activity induces the turnover of LVGCCs from the plasma membrane through endocytosis [34] and regulates the transcription of genes encoding for calcium-binding proteins and calcium-mediated signaling [44], mechanisms aiming to restore local Ca^2+^ signaling cascades and protect cells against aberrant Ca^2+^ influx. We show that BIN1 interacts with Cav_1.2_ in hiNs, similar to previous findings in cardiac T tubules [33] and provide evidence supporting a novel role for BIN1 in the regulation of activity-dependent internalization of Cav_1.2_ in human neurons, thus linking the known role of BIN1 in endocytosis [12] to firing homeostasis in human neurons via the LVGCC. These results confirm in human neurons the interaction between endogenous neuronal BIN1 and Cav_1.2_ as previously suggested in mouse hippocampal neurons overexpressing a Bin1-mKate fused protein [17].

Loss of Ca^2+^ homeostasis is an important feature of many neurological diseases and has been extensively described in AD [46, 47]. Interestingly, DEGs identified in *BIN1* KO glutamatergic neurons in our different cell culture models are enriched for calcium-related biological processes. This is also observed for DEGs detected in glutamatergic neurons of the AD brain at late stages of pathology when the expression levels of *BIN1* in those cells is also decreased. Thus, it is plausible to speculate that reduced expression of *BIN1* in glutamatergic neurons may contribute to the breakdown of Ca^2+^ homeostasis in the AD brain, potentially contributing to neuronal circuit dysfunctions. Consistent with this hypothesis, we have previously shown a significant reduction in the expression of the transcript encoding for the neuron-specific BIN1 isoform 1 in bulk RNA-sequencing data from a large number of AD patients [7] and we show in this work that *BIN1* expression is reduced in glutamatergic neurons of AD brains at late Braak stages. Moreover, it has been recently shown that *Bin1* conditional KO in neurons and glial cells of the mouse forebrain is sufficient to elicit gene expression changes associated with calcium-dependent mechanisms [48], further supporting the interpretation that BIN1 plays an important role in cellular processes involved in Ca^2+^ homeostasis in the brain.

Lastly, we show that treatment with the clinically approved calcium channel blocker nifedipine for only 2 weeks is sufficient to partly recover electrical and gene expression alterations in *BIN1* KO hiNs. These findings further support our interpretation that changes in gene expression and electrical activity observed in *BIN1* HET and KO hiNs are a direct consequence of reduced *BIN1* expression in glutamatergic neurons and not a possible artifact of CRISPR/Cas9 gene-editing in our hiPSC lines. Moreover, together with our observations of reduced *BIN1* expression and transcriptional alterations affecting biological processes related to calcium homeostasis in human-induced glutamatergic neurons of the human brain at late stages of AD pathology, our data strongly support a link between *BIN1* and calcium homeostasis.

Thus, it is plausible to speculate that reduced *BIN1* expression in glutamatergic neurons primarily undermines Ca^2+^ homeostasis, leading to changes in neuronal electrical activity. At a later stage, gene expression and circuit-level alterations such as synapse loss would occur, likely because of altered neuronal electrical activity. A corollary to this model would be that early treatments aiming to restore Ca^2+^ homeostasis and neuronal electrical activity may have a beneficial impact in AD onset and progression. Supporting this notion, both a Mendelian randomization and a retrospective population-based cohort study found evidence suggesting that treatment with Ca^2+^ channel blockers in human patients are associated with a reduced risk of AD [49, 50]. In the future, it would be interesting to study the impact of these drugs for AD onset/progress as a function of genetic variants in the *BIN1* locus.

An important limitation of our work is the absence of microglial cells in our models, hampering the study of *BIN1* roles in this cell type of high relevance to AD pathology [51]. However, our findings provide fundamental information about the molecular and cellular processes impacted by reduced *BIN1* expression in human neurons, which will require further confirmation in the brain of patients carrying AD-related *BIN1* genetic variants. Moreover, we studied the impact of *BIN1* HET and KO mutations, which do not necessarily represent the consequences of AD-related *BIN1* genetic variants in gene expression. Nevertheless, it is tempting to speculate that slight changes in *BIN1* expression provoked by those variants could progressively deteriorate neuronal functions in the human brain, contributing to AD pathogenesis in the elderly brain.

## Supplementary information

Supplementary Material

Supplementary Table 1

Supplementary Table 2

Supplementary Table 3

Supplementary Table 4

Supplementary Table 5

Supplementary Table 6

Supplementary Table 7

Supplementary Table 8

Supplementary Table 9

Supplementary Table 10

Supplementary Movie 1

Supplementary Movie 2

Supplementary Movie 3

Supplementary Movie 4

## Data Availability

Single-cell transcriptomic data is available at Mendeley Data doi: 10.17632/b3rf6fbjys.2. Supplementary information is available at MP’s website.
