# Supplementary material for: The Alzheimer’s disease risk gene BIN1 regulates activity-dependent gene expression in human-induced glutamatergic neurons
Source: Mol Psychiatry. 2024 Mar 22;29(9):2634–46. doi: 10.1038/s41380-024-02502-y (PMC11420064; doi:10.1038/s41380-024-02502-y)
Supplement: Supplementary file 1 — Supplementary Material [file 41380_2024_2502_MOESM1_ESM.pdf]

The Alzheimer's disease risk gene *BIN1* regulates activity-dependent gene expression in human-induced glutamatergic neurons

Supplementary information

1. Supplementary methods

1.1. *Maintenance of isogenic hiPSC lines and neural induction*

Isogenic hiPSCs (ASE 9109, Applied StemCell Inc. CA, USA) modified for *BIN1* in exon 3 were generated by CRISPR/Cas9 by the same company. Homozygous null mutant/knockout (KO) for *BIN1* had a 5 bp deletion on one allele and an 8 bp deletion on the other allele. Heterozygous (HET) for *BIN1* had a 1bp insertion on one allele. A clone subjected to CRISPR/Cas9 but showing no modification on the BIN1 locus was selected and used as wild-type (WT) control. Complete reports for the 3 main milestones during CRISPR/cas9 gene editing are provided at the end of this document. We used the IDT (Integrated DTA Technologies, Inc.) CRISPR-Cas9 guide RNA design checker (https://eu.idtdna.com/site/order/designtool/index/CRISPR_SEQUENCE) to evaluate potential off-targets of the guide RNA used for the generation of *BIN1* WT, HET and KO hiPSC clones. We identified only 6 possible target genes with 3 or 4 mismatches and a low potential of editing (Supplementary Table 1). Nevertheless, we performed the whole genome sequencing of all 3 hiPSC lines and confirmed the absence of off-targets effects in all possible off-target regions, as well as the normal ploidy of the cells (Supplementary Figure 1 and Supplementary Table 1). hiPSCs were maintained in mTeSR1 medium in non-treated cell culture dishes/plates pre-coated with vitronectin XF diluted in CellAdhere (Stemcell Technologies). Cells were passed when reached around 80% of confluency. Cell numbers and viability were recorded using a LUNA™ Automated Cell Counter.

To generate *BIN1* WT and KO human induced neural progenitor cells (hiNPCs), we used the dual SMAD-inhibition (SMADi) monolayer method (Chambers et al., 2009) and three independents neural inductions were done. Briefly, hiPSCs were cultured at high density (2x106 cells/well in a 6 wells plate) in cell culture dishes pre-coated with poly-L-ornithine (PLO) (15 ug/mL) and laminin (5 µg/mL) and passaged for 3 times in STEMdiff™ Neural Induction Medium + SMADi (Stemcell Technologies) for up to 3 weeks with daily full medium change. After this period, hiNPCS maintained for up to 10 passages in treated cell culture dishes pre-coated with poly-L-ornithine (PLO) (15 ug/mL) and laminin (5 µg/mL), using

STEMdiff™ Neural Progenitor Medium (NPM; Stemcell Technologies). PLO solution was made in PBS 1X water (0.001%) while laminin was diluted in PBS with Ca^{2+} and Mg^{2+}.

1.2. *Generation of cerebral organoids*

Cerebral organoids (3D Cultures) were generated from BIN1 WT, HET and KO hiPSCs using a 4-stage protocol (Lancaster et al., 2013) using the STEMdiffTM Cerebral Organoid Kit (Stemcell Technologies). The first step consists of the Embryoid Body (EB) Formation Stage, where hiPSCs at 80%-90% confluency were detached from the Vitronectin XF substrate using Accutase (#AT-104, Innovative Cell Technologies) to form the EB. 9000 cells were then plated per well in a 96-well round-bottom ultra-low attachment plate containing EB seeding medium (Stemcell Technologies). At day 2 and day 4, EB formation media was added in each well. After five days, 2 EBs were transferred per well in a 24-well ultra-low attachment plate containing Induction Medium (Stemcell Technologies). Two days later, the EBs were ready for the Expansion Stage. The EBs were embedded in a 16 ml drop of Matrigel (Corning) and transferred to a 6-well ultra-low attachment plate with Expansion Medium (Stemcell Technologies). After three days, the medium was replaced by Maturation Medium (Stemcell Technologies) and the plate was placed in an orbital shaker (65 rpm speed). During this final Maturation Phase, 75% medium change was done on a biweekly basis. Organoids were allowed to mature for a period of 6.5 months.

1.3. Generation of pure human neuronal cultures from hiNPCs

We differentiated neurons from virus-transduced hiNPCs according to a new protocol adapted from Yang et al. [1]. Briefly, hiNPCs were first virally transduced with a lentiviral construct encoding for the rtTA under control of a UbC promoter (Vect'UB; ID # 571) and one passage later, a second lentiviral construct encoding for Ascl1 and PuroR under the control of a TetO promoter (Addgene, # 97329). These cells were maintained in NPM medium and expanded prior to differentiation. For differentiation of human induced neurons (hiNs), hiNPCs were plated onto PLO/laminin-coated imaging plates (bottom 25 µm film, Eppendorf®) at density 50,000 cells/cm2 in NPM. After 24 h, complete BrainPhys medium (Stemcell Technologies) was added 1:1 together with 2 µg/mL doxycycline (Sigma-Aldrich) to induce TetO-regulated Ascl1 and PuroR expression. The following day, 1 µg/mL puromycin (Sigma-Aldrich) was added to start cell selection. After 2-3 days (depending on the efficiency of antibiotic selection), full medium change was performed, and 50,000 human cortical astrocytes (see below) were added in each well with BrainPhys containing doxycycline. After 24 h, 2 µM

of Cytosine β-D-arabinofuranoside (Ara-C; Sigma-Aldrich) was added to arrest the proliferation of astrocytes. Half of the medium in each well was changed biweekly with fresh BrainPhys medium containing doxycycline until the 14th day. After that, the biweekly medium change was performed only with complete BrainPhys. Differentiation was allowed to continue for another 2-4 weeks prior to subjecting the cells to various experimental manipulations.

Human cortical astrocytes (HA; ScienCell) were sourced from ScienCell Research Laboratories, CA, USA and grown up to 10 passages on poly-l-lysine coated 75 cm2 flasks using astrocyte medium (ScienCell) supplemented with astrocyte growth supplement (AGS, ScienCell) and 10 ml of fetal bovine serum (ScienCell).

This co-culture system was characterized using snRNA-seq after 4 weeks of differentiation showing that 70% of cells (n=7120 from 5 independent culture batches) expressed the pan-neuronal markers SOX11, SNAP25, DCX and RBFOX3, with 66% of cells co-expressing the glutamatergic neuron marker SLC17A6, less than 1.5% of cells co- expressing the GABAergic neuron markers DLX1, GAD1 and GAD2, and 5% of cells co-expressing low levels of markers of both neuronal subtypes. The remaining cells, immature astrocytes (Astro-I), mature astrocytes (Astro-II) and undifferentiated NPCs, represented about 15%, 8%, 4% of the cells, respectively. The first two cell populations likely represent two different states of astrocytes added to the cultures, whereas NPCs are likely cells that failed to reprogram into hiNs despite ASCL1 transduction. Thus, neuronal classes generated in this system comprise a large fraction of glutamatergic neurons (84%), a small proportion of GABAergic neurons (2.5%) and unspecified neurons co-expressing low levels of markers of both neuronal subtypes (13.5%).

1.4. Culture of hiNs in microfluidic devices

Three-compartment microfluidic neuron culture devices were used in which the presynaptic and postsynaptic chambers are connected to the synaptic chamber by respectively long and short micro-channels. Details of the microfluidic device design and fabrication have been previously described [2].

The homemade devices were placed individually in Petri dishes for easy handling and UV sterilized for 30 min before coating for cell adhesion. The primary surface coating consisted of poly-L-lysine (Sigma-Aldrich) at 20 µg/mL in borate buffer (0.31% boric acid, 0.475% sodium tetraborate, pH 8.5). All coated devices were incubated overnight at 37°C, 5% CO2. After a wash with DPBS, devices were then coated with 20 µg/mL laminin in DPBS and incubated overnight at 37°C in 5% CO2. The following day, devices were carefully washed once with DPBS before cell plating.

In total, 30,000 hiNPCs resuspended in NPM containing 10 µM of Y-27632 ROCK inhibitor were seeded per device, half at the entrance of the presynaptic somatic chamber and half at the entrance of the postsynaptic somatic chamber. Microfluidic devices were microscopically checked at the phase contrast to ensure the cells were correctly flowing into chambers. After a minimum of 5 minutes to allow the cells to attach, devices were filled with NPM (containing 10 µM of Y-27632 ROCK Inhibitor). Water was added to the Petri dishes to prevent media evaporation, and these were then incubated at 37°C in a humidified 5% CO2 incubator. To induce Ascl1 expression in hiNPCs, doxycycline (2 µg/mL) was added on the first day of half medium change to induce TetO gene expression. The following day, puromycin (1 µg/mL) was added to start cell selection. Two days after the puromycin selection, a total of 5,000 HAs (ScienCell Research Laboratories, CA, USA) were added per device. After 24 hours, Ara-C (2 µM) was added to stop their proliferation. Half of the medium was changed twice a week with complete BrainPhys medium + 2 µg/mL doxycycline for 14 days. After that, half medium change was performed only with BrainPhys medium.

For the quantification of synaptic puncta, five microfluidic devices were employed for each experimental condition (*BIN1* KO vs WT) and two independent cultures were performed. To assess the time-course effect, neuron cultures were stopped at 4 and 6 weeks. For MEA recordings using the MEA2100-256-System (Multi-Channel Systems), microfluidic devices were bonded onto multi-electrode arrays (256MEA100/30iR-ITO, Multi-Channel Systems, Germany) and cell cultures (n=5 per genotype, from at least 2 independent culture batches) were performed as detailed above.

1.5. Viral transductions

Lentiviral constructs were produced by the Vect'UB platform within the TBM Core unit at University of Bordeaux, Bordeaux, France (CNRS UMS 3427, INSERM US 005). The lentiviral constructs used were TTA (Vect'UB; ID # 571) and TetO-Ascl1-Puro (Addgene, # 97329). Lentiviral infections were done in NPCs at P3 or P4. The viral constructs were transduced at a multiplicity of infection (MOI) of 2.5. In brief, NPCs were plated at a confluency of 1x106 cells per well of a 6-well plate. After 4 hours of plating the cells, appropriate volumes of each lentiviral construct were mixed in complete Neural Progenitor medium and 50 µl of the viral medium mix was then added to each well. We transduced the TTA construct at first in the hiNPCs. Following one passage, the TTA-transduced cells were transduced with the construct for ASCL1. Cells having both viral constructs were then further expanded for 1 or 2 passages before being used for differentiation into hiNs.

The iGluSnFR construct was an adeno-associated viral vector (BS11-COG-AAV8) sourced from Vigene Biosciences, MD, USA. The viral construct was transduced at a MOI of 5,000 at around 10 days of differentiation for the ASCL1-hiNs. Differentiation was allowed to continue for a duration of 4-6 weeks prior to MEA electrophysiology or imaging.

1.6. Immunocytochemistry and immunohistochemistry

Bidimensional (2D) cultures in 24-well plates:

All cells were pre-fixed in 4% (w/v) paraformaldehyde (Electron Microscopy Sciences for 5 minutes in the imaging plates, followed by fixation for 10 minutes. Once fixed, cells were washed thrice with PBS 0.1 M. Blocking solution (5% normal donkey serum + 0.1% Triton X-100 in PBS 0.1 M) was added to fixed cells at room temperature for 1 hour under shaking conditions. After the blocking step, primary antibodies were added to cells in the blocking solution and incubated overnight at 4°C. The following day, cells were washed with PBS 0.1 M thrice for 10 mins. Alexa Fluor®--conjugated secondary antibodies in blocking solution were then incubated with the cells for 2 hours at room temperature under shaking conditions ensuring protection from light. Subsequently, 3 washes with 0.1 M PBS were done for 10 min each at room temperature under shaking conditions with protection from light. Hoechst 33258 solution was added during the second PBS wash. Cells were mounted with Aqua-Poly/Mount (Polysciences, Inc.) and imaged directly in the cell imaging plates. All images were acquired using an LSM 880 Confocal Scanning Microscope housed at the Imaging Platform of the Pasteur Institute, Lille. Duolink® Proximity Ligation Assays (PLA) was used to detect endogenous Protein-Protein Interactions. Antibodies used are listed below.

Microfluidic Devices:

Cultured induced neurons were fixed in 4% paraformaldehyde in PBS for 15 min at room temperature, washed three times with PBS, and permeabilized with 0.3% Triton X-100 in PBS for 5 min at room temperature. Cells were blocked in PBS containing 5% normal donkey serum for 1 h at room temperature before overnight incubation at 4°C with the primary antibodies listed below. Cells were washed twice with PBS and incubated with the secondary antibodies listed below for 2h at room temperature. ~~-~~Cells were rinsed three times with PBS and microfluidic devices were mounted with 90% glycerol. Samples were imaged with a LSM 880 confocal microscope with a 63× 1.4 NA objective. Images were acquired at zoom 2 in z-stacks of 0.5 µm interval. Typically, 6 images were acquired per device from the synapse chamber near the postsynaptic chamber such the image contains multiple dendrites. Images were deconvoluted using the Huygens software (Scientific Volume Imaging, Netherlands).

Cerebral Organoids:

Cerebral organoids were fixed in 4% PFA (w/v) for 30 min at 4°C followed by three washes with PBS 0.1 M. Cerebral organoids were then placed in sucrose solution (30% w/v) overnight before being embedded in O.C.T (Tissue-Tek). Embedded tissue was sectioned at 20 µm using a Cryostar NX70 Cryostat (Thermo Scientific) and mounted slides were stored at −80°C until immunostaining was performed. For immunostaining, tissue sections were brought to room temperature and then rehydrated with 3 washes with 0.1 M PBS, each for 5 mins. Slides were then washed once with PBS with 0.2% Triton X-100 for 15 mins. Tissue was blocked using 10% of donkey serum in PBS 0.1 M for 1 h at room temperature. After blocking, primary antibodies were added to 0.2 % Triton X-100 and 10% of donkey serum in PBS 0.1 M at appropriate dilutions and incubated overnight at 4°C. The next day, slides were washed with PBS 0.1 M 3 times for 5 min each with gentle shaking. Subsequently, slides were incubated with Alexa Fluor®-conjugated secondary antibodies in 0.2 % Triton X- 100 and 10% of donkey serum in PBS 0.1 M for 2 h at room temperature in the dark. After secondary antibody incubation, slides were washed 3 times with PBS for 5 min with gentle shanking. Nuclei were visualized by incubating the tissue for 5 min with Hoechst 33258 stain in PBS 0.1 M. Sections were mounted using aqueous mounting medium (Polysciences). Images were acquired using an LSM 880 Confocal Scanning Microscope in concert with the ZEISS ZEN imaging software housed at the Imaging Platform of the Pasteur Institute, Lille.

List of antibodies used in this study

Antibody	Host species	Catalog no.	Source	IF	WB
APP C- terminal	Rabbit	A8717	Sigma-Aldrich		x
Beta III Tubulin	Mouse	MAB1637	Sigma-Aldrich	x	
BIN1	Rabbit	182562	Abcam	x	x
Blocking peptide for CaV1.2 (CACNA1C)	-	BLP-CC003	Alomone		x
Cav1.2	Mouse	84814	Abcam	x	
CaV1.2 (CACNA1C)	Guinea Pig	ACC-003	Alomone		x
Cav1.3	Mouse	85491	Abcam	x	
CaV1.3 (CACNA1D)	Rabbit	ACC-005	Alomone		x
CaV2.1 (CACNA1A)	Rabbit	ACC-001	Alomone		x

CaV2.2 (CACNA1B)	Rabbit	ACC-002	Alomone		x
CaV2.3 (CACNA1E)	Rabbit	ACC-006	Alomone		x
GFAP	Chicken	173006	Synaptic Systems	x	
GFAP	Rabbit	AB5804	Sigma-Aldrich	x	
GFAP	Rabbit	AB5804	Millipore	x	
HOMER1	Guinea pig	160004	Synaptic Systems	x	
hTAU-C	Rabbit	A0024	Dako		x
MAP2	Guinea pig	188004	Synaptic Systems	x	
MAP2	Chicken	188006	Synaptic Systems	x	
Phospho-TAU (AT8)	Mouse	MN1020	ThermoFisher Scientific		x
Synaptophysin	Mouse	101011	Synaptic Systems	x	
β-ACTIN	Mouse	A1978	Sigma-Aldrich		x
Alexa Fluor 488	Donkey Anti-Mo	715-545-151	Jackson ImmunoResearch	x	
Alexa Fluor 594	Donkey Anti-Gp	706-585-148	Jackson ImmunoResearch	x	
Alexa Fluor 647	Donkey Anti-Rb	711-605-152	Jackson ImmunoResearch	x	
DyLight™ 405	Donkey Anti-Ch	703-475-155	Jackson ImmunoResearch	x	
Guinea pig-HRP	Goat	106-035-003	Jackson ImmunoResearch		x
Mouse- HRP	Goat	115-035-003	Jackson ImmunoResearch		x
Rabbit-HRP	Goat	111-035-003	Jackson ImmunoResearch		x

1.7. Quantification of synaptic connectivity

Synaptic connectivity was quantified as previously described [2]. Briefly, images were analyzed with Imaris software (Bitplane, Zürich, Switzerland) by reconstructing Synaptophysin I and HOMER1 puncta in 3D. The volume and position information of all puncta were processed using a custom Matlab (MathWorks, Natick, MA) program. This program assigns each postsynaptic spot to the nearest presynaptic spot (within a distance threshold of 1 µm) and calculates the number of such assignments for all presynaptic puncta.

1.8. Immunoblotting

Samples from the 2D cultures or brain organoids were collected in RIPA buffer containing protease and phosphatase inhibitors (Complete mini, Roche Applied Science) and sonicated several times at 60%-70% for 10 seconds prior to use for the immunoblotting analyses. Protein quantification was performed using the BCA protein assay (ThermoFisher Scientific). 10 µg

of protein from extracts were separated in NuPAGE 4-12% Bis-Tris Gel 1.0mm (NP0321BOX, Thermo Scientific) or 3-8% Tri-Acetate gel (EA03755BOX, Thermo Scientific) and transferred on to nitrocellulose membranes 0.2µm (#1704158, Bio-Rad). Next, membranes were incubated in milk (5% in Tris-buffered saline with 0.1% Tween-20 (TBST)) or SuperBlock (37536, ThermoFisher Scientific) to block non-specific binding sites for 1 hour at room temperature, followed by several washes with TBST 0.1% or TNT 1x as washing buffers. Immunoblottings were carried out with primary antibodies overnight at 4°C under shaking condition. The membranes were washed three times in the washing buffer, followed by incubation with HRP-conjugated secondary antibodies for 2 hours at room temperature under shaking condition. The membranes were washed three times in washing buffer, and the immune reactivity was revealed using the ECL chemiluminescence system (SuperSignal, ThermoScientific) and imaged using the Amersham Imager 600 (GE Life Sciences). Optical densities of bands were quantified using the Gel Analyzer plugin in Fiji–ImageJ. The primary antibodies used for the immunoblots are listed on the antibody table (see above).

1.9. Activity-dependent endocytosis assay

ASCL1-hiNs (n=9 cultures from each genotype) were subjected to 30 min of depolarization with 65 mM KCl or a mock treatment. Cells were then collected and pulled for endosomal fraction purification using the Minute™ Endosome Isolation and Cell Fractionation Kit (Invent Biotechnologies). Western blot was performed as described above.

1.10. AlphaLISA measurements

Cell culture media samples for AlphaLISA measurements were collected at the endof the 3rd and 4th weeks of differentiation of the ASCL1-hiNs. Alpha-LISA kits specific for human Aβ1–X (AL288C, PerkinElmer) and Aβ1–42 (AL276C, PerkinElmer) were used to measure the amount of Aβ1–X and Aβ1–42 respectively in culture media. The human Aβ analyte standard was diluted in the BrainPhys medium. For the assay, 2 µL of cell culture medium or standard solution was added to an Optiplate-384 microplate (PerkinElmer). 2 µL of 10X mixture including acceptor beads and biotinylated antibody was then added to the wells with culture media or standard solution. Following incubation at room temperature for an hour, 16 µL of 1.25X donor beads was added to respective wells and incubated at room temperature for 1 hour. Luminescence was measured using an EnVision-Alpha Reader (PerkinElmer) at 680-nm excitation and 615-nm emission wavelengths.

1.11. Calcium and iGluSnFR imaging

Calcium imaging was performed in 2D cultures after 4 weeks (Ascl1-induced). Prior to imaging, the cells were incubated with Oregon Green™ 488 BAPTA-1 (OGB-1) acetoxymethyl (AM) (ThermoFisher Scientific) for 1 h. A 2.5 mM stock solution of the calcium-indicator dye was prepared in Pluronic™ F-127 (20% solution in DMSO) (ThermoFisher Scientific). 1 µL of the dye solution was added to 400 µL of fresh BrainPhys medium in each well of a 24-well cell imaging plate. Existing BrainPhys media from the wells of the plate was removed and kept aside while the calcium-indicator dye was incubated in fresh BrainPhys medium. After 1 h of incubation, the medium which was kept aside was replaced to each well. The 2D cultures were then ready to be imaged using a Spinning Disk Microscope (Nikon) housed at the Institut Pasteur de Lille, Lille, France using the MetaMorph imaging software. 1000 images were taken using a 20× long-distance objective, with 10 ms exposure time and 200 ms intervals (5 Hz sampling rate). For each well, 5 random fields were chosen, and the cellular activity was recorded.

Cells transduced with iGluSnFR were directly imaged after 4 or 6 weeks of differentiation and 500 images were taken using the same imaging parameters. Up to 8 fields per well were filmed, each field containing at least one fluorescent transduced cell along with its processes.

1.12. Analyses of calcium transients

All live recordings of neuronal calcium transients were first converted into .avi format after background subtraction using the FIJI software. Following the conversion, the videos were subsequently opened using the free software for data analyses of calcium imaging, CALciumIMagingAnalyzer (CALIMA) [3]. Each video recording of a field of cells was first downscaled to 2× in terms of size with a 10× zoom and was checked for the frame average mode. Moreover, in this first detection stage, pre-set filter parameters were adjusted and applied to enable the detection of the maximum number of fluorescent cells in each field. In the analysis tab, detection of the average activity was checked and for pre-processing, a median of 3 was applied. All cells within the pre-set filter parameters are detected as regions of interest (ROIs) in the detection stage. Cell activity from all detected ROIs is then recorded. However, in the subsequent analysis stage, only cells showing spiking frequencies with a standard deviation of at least 2 or more were taken into consideration. Data in the form of detection spikes and the correlation (peak) are extracted and exported as .csv files.

1.13. Electrophysiological recordings in 2D cultures and cerebral organoids

ASCL1-hiNs were cultured in microfluidic devices bound to multi-electrode arrays (256MEA100/30iR-ITO, Multi-Channel Systems, Germany). Extracellular action potentials were recorded in 5 different cultures for both genotypes at 2, 3, 4 and 6 weeks of differentiation using the MEA2100-256-System (Multi-Channel Systems). Before recordings, MEAs were let stabilize for 5 min on the headstage to reduce artifacts due to medium movement. Signals were recorded for 1 min, at 40 kHz sampling rate, using Multi Channel Experimenter 2.16.0 software (Multi-Channel Systems). For rescue experiments using a calcium channel blocker, ASCL1-hiNs were cultured on MEA 96-well plates (CytoView MEA 96, Axion Biosystems, USA). Extracellular action potentials were recorded in 3 independent cultures for either genotype in the presence of 50 nM nifedipine (Tocris Bioscience) or vehicle using the MaestroPro (Axion Biosystems, Inc, USA). Before recordings, MEAs were let stabilize for 5 min on the MaestroPro at 37ºC and 5% CO_2. Signals were recorded for 3 min, at 12.5 kHz sampling rate, using AxIS Navigator software (Axion Biosystems).

Spikes were detected using a fixed amplitude threshold of 5.5 standard deviations of the high-pass filtered (>300 Hz) signal for positive- and negative-going signals. The detection included a dead time of 3 ms to account for the refractory period of action potentials. Quantification of the number of detected spikes (MUAs) and spike bursts (defined as at least 5 spikes within 50 ms) was performed using Multi-Channel Analyzer 2.16.0 software (Multi-Channel Systems).

1.14. Spike sorting and temporal structure of spontaneous activity

Channels containing detected waveforms were manually processed offline for spike waveform separation and classification using Offline Sorter v3 (Plexon, USA). Briefly, we applied principal component analysis (PCA) to cluster spike waveforms of similar morphologies. Using this approach, we identified from 2 to 10 well-isolated units per channel, and therefore, we considered this single-unit activity (SUA). For each SUA, we computed the average firing rate, the signal-to-noise ratio, the peak-to-trough amplitude and duration, the average power (square amplitude of the average waveform), the mode of the interspike interval distribution, and their firing patterns. It has been demonstrated that dissociated neuronal cultures can develop complex discharge structures [4, 5]. Here, we considered burst activity if the SUA presents periods of high-frequency discharges interspersed by regular or no discharges at all. Operationally, a burst must have at least 3 spikes within 100 ms and 200 ms intervals, for the interval between the first and the second, and the second and the third discharge, respectively. After the third spike, the maximal interval to consider a discharge part of the burst

was 200 ms. Thus, we computed the SUA that presented bursts, the number of bursts (i.e., the burst frequency), the average burst duration, the number of spikes within each burst, the average burst frequency, and the inter-burst interval. To evaluate the temporal structures of spike trains, we computed the array-wide spike detection rate (ASDR), which is the number of spikes detected per unit of time, summed over all electrodes in the array. This method is commonly used in the literature to demonstrate synchronous activity (aka, bursts) in MUA data [4].

1.15. Whole genome sequencing

DNA Libraries was prepared using BGI sequencing workflow (ref). Reads were cleaned using Soapnuke [6]. Reads was then aligned to the hg38 genome using bwa-0.7.17 [7], converted to BAM file and sorted using samtools [8]. For each sample, read duplicates was masked using MarkDuplicatesSpark module from gatk tool [9], and variants detected in the predicted gBIN1 binding regions (in a +/- 10bp window) was called using gatk HaplotypeCaller in GVCF mode specifying these intervals. Generated GVCF files was merge and definitive genotype was called using CombineGVCFs and GenotypeGVCFs tool. Variants was filtered according to these QC parameters: QD<2, QUAL < 50, SOR > 3.0, MQ < 40.0, MQRankSum < -12.5, and ReadPosRankSum < -8.0 for SNPs; and QD<2, QUAL < 30, FS > 200, MQ < 40.0 and ReadPosRankSum < -20.0 for INDELs.

To identify putative aneuploidy, nQuire tool was used [10]. From reads with mapping quality more than 30, base frequencies at variables sites were measured and Gaussian mixture model was used to estimate the ploidy level for each chromosome in each sample. The root means square error (RMSE) between the fixed ploidic model, and the free model is used to evaluate which ploidy model fit the most with the observed distribution.

1.16. snRNA-seq library preparation

Nuclei isolation and Hash-tagging with oligonucleotides steps were realized on ice with pre-cold buffers and centrifugations at 4°C. 6.5-month-old BIN1 WT, HET, and KO organoids (n=4 from each genotype) were processed as previously [11]. 4-week-old cultured ASCL1-induced BIN1 WT and KO 2D cultures (n=5 independent culture batches) were washed in the imaging plate wells with 500 µL of Deionized Phosphate Buffer Saline 1X (DPBS, GIBCO™, Fisher Scientific 11590476). Cells were resuspended with wide bore tips in 500 µL Lysis Buffer (Tris-HCL 10mM, NaCl 10mM, MgCl2 3mM, Tween-20 0,1%, Nonidet P40 Substitute 0,1%, Digitonin 0,01%, BSA 1%, Invitrogen™ RNAseout™ recombinant ribonuclease

inhibitor 0,04 U/µL). Multiple mechanical resuspensions in this buffer were performed for a total lysis time of 15 mins., 500 µL of washing buffer was added (Tris-HCL 10mM, NaCl 10 mM, MgCl2 3 mM, Tween-20 0.1%, BSA 1%, Invitrogen™ RNAseout™ recombinant ribonuclease inhibitor 0,04 U/µL) and the lysis suspension was centrifuged 8 mins. at 500 g (used for all following centrifugation steps). Nuclei pellets were washed tree times with one filtration step by MACS pre-separation filter 20µm (Miltenyi Biotec). Nuclei pellets were resuspended in 100 µL of staining buffer (DPBS BSA 2%, Tween-20 0.01%), 10 µL of Fc blocking reagent HumanTruStainFc™ (422302, Biolegend) and incubated 5 min at 4°C. 1µl of antibody was added (Total-Seq™-A0453 anti-Vertebrate Nuclear Hashtag 3 MAb414 for the WT and Total-Seq™-A0454 anti-Vertebrate Nuclear Hashtag 4 MAb414 for the KO, 97286 and 97287 respectively, Biolegend) and incubated 15 mins. at 4°C. Nuclei pellets were washed three times in staining buffer with one filtration step by MACS pre-separation filter 20 µm (Miltenyi Biotec) to a final resuspension in 300 µL of staining buffer for Malassez cell counting with Trypan blue counterstaining (Trypan Blue solution, 11538886, Fisherscientific). Isolated nuclei were loaded on a Chromium 10X genomics controller following the manufacturer protocol using the chromium single cell v3 chemistry and single indexing and the adapted protocol by Biolegend for the HTO library preparation. The resulting libraries were pooled at equimolar proportions with a 9 for 1 ratio for Gene expression library and HTO library respectively. Finally, the pool was sequenced using 100pb paired end reads on NOVAseq 6000 system following the manufacturer recommendations (Illumina).

1.17. snRNA-seq dataset preprocessing

Unique Molecular Index (UMI) Count Matrices for gene expression and for Hash Tag Oligonucleotide (HTO) libraries were generated using the CellRanger count (Feature Barcode) pipeline. Reads were aligned on the GRCh38-3.0.0 transcriptome reference (10x Genomics). Filtering for low quality cells according to the number of RNA, genes detected, and percentage of mitochondrial RNA was performed. For HTO sample, the HTO matrix was normalized using centered log-ratio (CLR) transformation and cells were assigned back to their sample of origin using HTODemux function of the Seurat R Package (v4) [12]. Then, normalizations of the gene expression matrix for cellular sequencing depth, mitochondrial percentage and cell cycle phases using the variance stabilizing transformation (vst) based Seurat:SCTransform function were performed.

1.18. snRNA-seq datasets integration and annotation

To integrate the datasets from independent experiments, the harmony R package (https://github.com/immunogenomics/harmony) was used. In order to integrate the datasets, the SCTransform normalized matrices was merged and PCA was performed using Seurat::RunPCA default parameter. The 50 principal components (dimensions) of the PCA were corrected for batch effect using harmony::RunHarmony function. Then, the 30 first batch corrected dimensions were used as input for graph-based cell clustering and visualization tool. Seurat::FindNeighbors using default parameters and Seurat::FindClusters function using the Louvain algorithm were used to cluster cells according to their batch corrected transcriptomes similarities. To visualize the cells similarities in a 2-dimension space, the Seurat::RunUMAP function using default parameter was used. Cell clusters were then annotated based on cell type specific gene expression markers.

1.19. *Differential gene expression and GO enrichment analyses*

For snRNA-seq experiment reproduce 2 times or less (*i.e.* organoid and nifedipine experiments), gene expression within each main cell type was compared between conditions of interest using Wilcoxon test on the SCTransform normalized gene expression matrix. For snRNA-seq experiment reproduce more than 2 times (*BIN1* KO vs WT hiNs comparison), Pseudobulk differential expression analysis was performed using the package DESeq2 (CRAN). For each main cell type, the cell level gene expression matrix was first aggregate at the sample level using Matrix::aggregate.Matrix function. Genes detected in more than 100 cells or in 10% of the cells was test for differential expression using DESeq function, including in the model the experiment batch as covariate.

GO enrichment analysis on the differentially expressed genes was performed using the gost function of the gprofiler2 R package (CRAN), using default parameter, or, if specified using fast gene-set enrichement analysis (FGSEA, [13]) package (Bioconductor).

1.20. Activity-related genes (ARGs) signature enrichment analysis at single cell resolution

To study enrichment for activity-related genes (ARGs) signature across cerebral organoid cells, the CellID R package (https://bioconductor.org/packages/release/bioc/html/CelliD.html) was used. ARGs obtained from Tyssowski et al. (2018) and Hravtin et al. (2018) [9, 10]. In the first work, Tyssowski et al. stimulated neurons with brief or sustained patterns of electrical activity and performed targeted ARG-seq experiments at different time-points after stimulation. Differentially expressed genes ($FDR<0.05$ and $FC>1.5$) were identified using

EdgeR. Gene lists were classified as primary response genes (PRGs) if they showed less than a 2-fold reduction in expression in 6h-KCl-treated neurons in the presence of of the translation-inhibitor cycloheximide, and secondary response genes (SRGs) if they showed a reduction greater than 2-fold in the presence of cycloheximide (FDR<0.05 by edgeR). PRGs were subclassified as rapid if they had higher induction at 1 h compared to 6 h and delayed if they had higher induction at 6 h compared to 1 h. All rapid PRGs showed >2-fold pre-mRNA induction by 20 min of stimulation. In the second work, Hrvatin et al. performed scRNAseq of mouse primary visual cortices after exposure to light for 1 h or 4 h before euthanasia. Differential gene expression analysis was performed in Monocle2 (Trapnell et al) for excitatory and inhibitory neurons independently. Genes whose FDR was < 0.05 and whose log2 fold change in expression was either > 1 or < −1 were considered ARGs. For each cell type, ARGs were classified as either ERGs or LRGs based on their expression patterns. If an induced gene's maximum expression was at 1 h, the gene was classified as an early-response gene (ERG), whereas if the maximum expression was at 4 h, the gene was classified as a late-response gene (LRG). The complete list of ARGs identified in both studies are available as supplementary material linked to the original publications. A summarized list is presented in this study (Supplementary table 3).

CellID::RunMCA was used to extract cell-specific gene signature and hypergeometric test was performed to test enrichment for ARGs in these cell signatures. To test the differential proportion of ARGs enriched cells in BIN1 deleted organoid compared to WT organoid, chi-squared test was performed. To evaluate the enrichment of ARGs in DEGs of nifedipine treated cells, overlap and hypergeometric test was performed.

1.21. Comparative analysis with specific DEGs in AD brains

To compare the transcriptomic change observed in BIN1 deleted cerebral organoid with those observed in AD brain we used the dataset available for download at Synapse.org under the Synapse ID syn21788402 [16]. The raw gene expression matrix was normalized using Seurat::SCTransform and differential expression analysis was performed within each neuronal cell type using Wilcoxon test as used for our organoid dataset. AD related DEGs, thus, obtained were compared with our BIN1 related organoid DEGs in every cell type. To this end, the enrichment for AD-related DEGs in BIN1-related DEGs was tested using hypergeometric test. The background for this test was defined as all genes detected in both datasets. The p-value of this test was used as metrics to compare the significance of the gene overlap between neuronal cell types.

2. Supplementary data

Sup. Figure 1: Whole genome Sequencing confirms absence of Off targets mutation and aneuploidy. (A) Table showing the mutations detected within predicted off target regions of the guide RNA used for CRISPR/Cas9 gene editing in BIN1 WT, HET and KO hiPSC clones. Complete list of predicted off target regions and the full variant calling results for these regions are available in Supplementary Table 1. (B) Barplot of the ploidy level evaluation using nQuire Tool. The y axis represents the root means square error (RMSE) between the fixed ploidic model, and the free model is used to evaluate which ploidy model fit the most with the observed distribution. Small values indicate the ploidy model most like the real distribution.

Sup. Figure 2: Cellular and molecular characterization of 6.5-month-old cerebral organoids. (A) Immunohistochemistry for GFAP (red), MAP2 (green) and DAPI (blue) in 6.5-month-old *BIN1* WT, HET and KO COs. (B) Western blots showing the isoforms of BIN1 detected in WT, WT, the reduced expression in HET, and the absence of BIN1 protein in KO COs. (C) Western blots showing the expression of neuronal (TAU and vGLUT2) and astrocytic (GFAP) proteins in 14 individual COs at 190 days, including some of the BIN1 WT, HET and KO samples used for snRNA-seq. Note the low variability in protein expression.

Sup. Figure 3: Comparison of gene expression alterations in *BIN1* HET and KO cerebral organoids. (A) Volcano plots representing DEGs comparing HET vs WT or KO vs WT in astrocytes and glutamatergic neurons. DEGs with adjusted p-value <0.05 and |log2FC| >0.25 are shown in red. Gene labels are shown for top 10 genes in terms of log2FoldChange and p-value. (B) Heatmap showing log2FC for all DEGs identified in GluNeu when comparing *BIN1* KO vs WT or HET vs WT. (C) Venn diagram showing the intercept between DEGs in GluNeu of *BIN1* HET and KO compared to WT COs. (D) Correlation between gene expression alterations observed in GluNeu and Astrocyte in the same comparisons. (E) Functional enrichment analysis showing the top 15 significantly enriched GOs for DEGs identified in the *BIN1* KO vs WT or HET vs WT glutamatergic neurons. GO: gene ontology; BP: biological processes; CC: cellular components; MF: molecular function.

Sup. Figure 4: Reduced BIN1 expression does not enhance activity-dependent transcription in GABAergic neurons. A-B) Proportions of GABAergic neurons enriched for

the different ARG signatures according to genotype (*p<0.05; **p<0.01; ***p<0.001; Chi-squared test).

Sup. Figure 5: Normal Tau phosphorylation and APP processing in 190 days-old *BIN1* HET and KO cerebral organoids. (A) Western blot for total TAU protein C-terminal (TAU-C), phosphorylated (p)-TAU at Ser202, Thr205 (AT8) and β-ACTIN in 6.5-month-old COs. (B-D) Quantification of TAU-C/β-ACTIN, p-TAU/β-ACTIN and p-TAU/TAU-C levels normalized to WT ($^{\#}p<0.1$; Mann-Whitney test; n=3 COs per genotype). (E) Western blot for APP and β-ACTIN in 6.5-month-old COs. (F-H) Quantification of APP/β-ACTIN, β-CTF/β-ACTIN and β-CTF /APP levels normalized to WT (n=3 COs per genotype). The uncropped blots used for this figure are shown in Sup. Figures 9, 10 and 11.

Sup. Figure 6: Comparison of gene expression alterations in Ascl1-hiNs and GluNeu in COs. (A) Overlap between DEGs identified in GluNeu of Ascl1-hiNs cultures (KO vs WT) and COs (KO vs WT; HET vs WT). (B) Heatmap showing GO terms enriched in KO vs WT DEGs at p-value <0.001 in at least one model.

Sup. Figure 7: *BIN1* KO ASCL-hiNs have a higher spike burst frequency compared to WT. (A) Raster plots showing MUA recorded for 1 minute in *BIN1* WT and KO ASCL1-hiNs after 4 weeks of differentiation. Each line represents one electrode localized side-by-side in our microfluidic/MEA array (as in panel A). (B-C) Quantification of the number of detected spikes at different time points (*p=0.0141; ***p=0.0006; Two-way ANOVA followed by Tukey's multiple-comparison test; n= 5 for each genotype). (D) Quantification of the number of spike bursts at different time points (**p=0.004; $^{\#}p=0.0888$; Mann-Whitney test).

Sup. Figure 8: Normal glutamatergic transmission in *BIN1* KO ASCL1-hiNs. Box plots show the number of active spots per neuron and number of events detected by time-lapse video-microscopy in 4- or 6-week-old ASCL1-hiNs cultures transduced with the glutamate sensor iGLUSnFr (4 weeks: n= 378 BIN1 WT and 266 BIN1 KO ASCL1-hiNs; 6 weeks: n= 685 BIN1 WT and 629 BIN1 KO ASCL1-hiNs).

Sup. Figure 9: Normal APP processing in *BIN1* KO ASCL1-hiNs. (A) Western blots showing the expression of APP full-length, β-CTF and β-ACTIN at 4 weeks. (B) Quantification of the ratios APP/β-ACTIN, β-CTF /β-ACTIN and β-CTF/APP (n = 5 for each genotype). (C)

Quantification of soluble $A\beta_{1\text{-}x}$, $A\beta_{1\text{-}42}$ and the ratio $A\beta_{1\text{-}42}/A\beta_{1\text{-}x}$ in ASCL1-hiNs cultures at 3 and 4 weeks.

Sup. Figure 10: Expression of voltage-gated calcium channels in ASCL1-hiNs. (A) Violin plots showing the mRNA levels of Cav1 and Cav2 members of the voltage-gated calcium channel families L-type, P/Q-type, N-type and R-type detected in ASCL1 hiNs. (B) Images showing PLA spots using anti-BIN1 and anti-Cav1.3 antibodies in 4-week-old *BIN1* WT hiNs. Cells were also immunolabeled for the neuronal marker MAP2 (green), the astrocyte marker GFAP (white), and stained with DAPI (blue). (C) Western blots showing the expression of Cav1.3, Cav2.1, Cav2.2, Cav2.3 and β-ACTIN in 4-week-old ASCL1h hiNs. (D) Quantification of protein expression.

Sup. Figure 11: snRNAseq of nifedipine treated ASCL1-hiNs cultures. (A) UMAP of the n=2 nifedipine experiments integrated with the other hiNs snRNA-seq experiments. (B) Barplot showing the total number of DEGs identified in *BIN1* KO ASCL1-hiNs treated or not with nifedipine. (C) Volcano plots showing differential gene expression in nifedipine treated vs untreated *BIN1* KO or WT GluNeu-II cells. Genes belonging to the LRGs Signature which are differentially expressed (adjusted p-value<0.05) are labeled. (D) Heatmaps of the enrichment for the different ARGs signature in DEGs up or downregulated in nifedipine treated condition in KO and WT GluNeu-II cells. (B) correlation between the differential gene expression in untreated *BIN1* KO + vehicle vs WT (x axis, DEGs are plotted in red) and in *BIN1* KO + nifedipine (nif) vs WT. Calcium- and synapse-related genes upregulated in *BIN1* KO + vehicle vs WT are labeled if the genes are not upregulated in KO + nif vs WT.

Sup. Figure 12: Full Western blots of *BIN1* WT and KO ASCL1-hiNs. Western blot for total TAU protein C-terminal (hTAU-C), phosphorylated (p)-TAU at Ser202, Thr205 (AT8) and β-ACTIN in 4-week-old ASCL1-hiNs cultures. The blots are identical to the ones shown in Fig. 5E but show the whole membranes.

Sup. Figure 13: Full Western blots of *BIN1* WT and KO ASCL1-hiNs. Western blot for Cav1.2 in the absence or presence of specific blocking peptide and β-ACTIN in 4-week-old ASCL1-hiNs cultures. The blots are identical to the ones shown in Fig. 6H but show the whole membranes.

Sup. Figure 14: Full Western blots of *BIN1* WT and KO ASCL1-hiNs. Western blot for Cav1.2 and β-ACTIN in total protein extracts, Cav1.2, Cav1.3 and EEA1 in the endosomal protein fractions obtained from 4-week-old ASCL1-hiNs cultures. The blots were cropped and reordered to show *BIN1* WT and KO genotypes in Fig. 6J-K.

Sup. Movies 1 and 2: Time-series of 1000 frames taken from *BIN1* WT and KO ASCL1-hiNs transduced with iGLUSnFr after 2 weeks of differentiation and imaged 2 weeks later. Videos are played at 100 frames per second (fps).

Sup. Movies 3 and 4: Time-series of 1000 frames taken from *BIN1* WT and KO ASCL1-hiNs after 4 weeks of differentiation and labeled with Oregon Green BAPTA and imaged. Videos are played at 100 fps.

Sup. Table 1: Variants identified in predicted regions target of gRNA sequence using Whole Genome Sequencing.

Sup. Table 2: DEGs identified in different cell types/subtypes of COs.

Sup. Table 3: GO terms enriched for DEGs identified in different cell types/subtypes of COs.

Sup. Table 4: List of ARGs used for hypergeometric test in Cell-ID.

Sup. Table 5: DEGs identified in different cell types/subtypes of ASCL1-hiNs cultures.

Sup. Table 6: GO terms enriched for DEGs identified in different cell types/subtypes of ASCL1-hiNs cultures.

Sup. Table 7: DEGs identified in different cell types/subtypes of ASCL1-hiNs cultures treated with nifedipine.

Sup. Table 8: GO terms enriched for DEGs identified in different cell types/subtypes of ASCL1-hiNs cultures treated with nifedipine.

Sup. Table 9: DEGs identified in different cell types/subtypes of the AD brain.

Sup. Table 10: GO terms enriched for DEGs commonly identified in *BIN1* HET or KO cells and the AD brain.

References

1. Yang N, Chanda S, Marro S, Ng YH, Janas JA, Haag D, et al. Generation of pure GABAergic neurons by transcription factor programming. Nat Methods. 2017;14:621–628.
2. Kilinc D, Vreulx A-C, Mendes T, Flaig A, Marques-Coelho D, Verschoore M, et al. Pyk2 overexpression in postsynaptic neurons blocks amyloid β1-42-induced synaptotoxicity in microfluidic co-cultures. Brain Commun. 2020:1–18.
3. Radstake FDW, Raaijmakers EAL, Luttge R, Zinger S, Frimat JP. CALIMA: The semi-automated open-source calcium imaging analyzer. Comput Methods Programs Biomed. 2019;179.
4. Wagenaar DA, Pine J, Potter SM. An extremely rich repertoire of bursting patterns during the development of cortical cultures. BMC Neurosci. 2006;7:1–18.
5. Plumbly W, Brandon N, Deeb TZ, Hall J, Harwood AJ. L-type voltage-gated calcium channel regulation of in vitro human cortical neuronal networks. Sci Rep. 2019;9.
6. Chen Y, Chen Y, Shi C, Huang Z, Zhang Y, Li S, et al. SOAPnuke: a MapReduce acceleration-supported software for integrated quality control and preprocessing of high-throughput sequencing data. Gigascience. 2018;7:1–6.
7. Li H, Durbin R. Fast and accurate short read alignment with Burrows–Wheeler transform. Bioinformatics. 2009;25:1754–1760.
8. Danecek P, Bonfield JK, Liddle J, Marshall J, Ohan V, Pollard MO, et al. Twelve years of SAMtools and BCFtools. Gigascience. 2021;10:giab008.
9. Poplin R, Ruano-Rubio V, DePristo MA, Fennell TJ, Carneiro MO, Auwera GA Van der, et al. Scaling accurate genetic variant discovery to tens of thousands of samples. BioRxiv. 2018:201178.
10. Weiß CL, Pais M, Cano LM, Kamoun S, Burbano HA. nQuire: a statistical framework for ploidy estimation using next generation sequencing. BMC Bioinformatics. 2018;19:122.
11. Lambert E, Saha O, Soares Landeira B, Melo de Farias AR, Hermant X, Carrier A, et al. The Alzheimer susceptibility gene BIN1 induces isoform-dependent neurotoxicity through early endosome defects. Acta Neuropathol Commun. 2022;10:4.
12. Hao Y, Hao S, Andersen-Nissen E, Mauck WM, Zheng S, Butler A, et al. Integrated analysis of multimodal single-cell data. Cell. 2021;184:3573-3587.e29.
13. Korotkevich G, Sukhov V, Sergushichev A. Fast gene set enrichment analysis. bioRxiv. BioAr. 2019. 2019.

14. Tyssowski KM, DeStefino NR, Cho JH, Dunn CJ, Poston RG, Carty CE, et al. Different Neuronal Activity Patterns Induce Different Gene Expression Programs. Neuron. 2018;98:530-546.e11.
15. Hrvatin S, Hochbaum DR, Nagy MA, Cicconet M, Robertson K, Cheadle L, et al. Single-cell analysis of experience-dependent transcriptomic states in the mouse visual cortex. Nat Neurosci. 2018;21:120–129.
16. Leng K, Li E, Eser R, Piergies A, Sit R, Tan M, et al. Molecular characterization of selectively vulnerable neurons in Alzheimer's disease. Nat Neurosci. 2021;24:276–287.

Figure S1

A

#CHROM	POS	REF	ALT	QUAL	FILTER	BIN1_HETA	BIN1_KO	BIN1_WTA
chr1	3896146	T	C	3103.73	PASS	1/1	1/1	1/1
chr2	88134245	A	G	4782.73	PASS	1|1	1/1	1/1
chr10	49386463	C	A	4667.73	PASS	1/1	1/1	1/1
chr10	76999151	T	G	1825.97	PASS	0/1	0/1	0/1
chr19	53441997	A	T	1235.97	PASS	0/1	0/1	0/1
chr20	29438421	A	G	819.97	PASS	0/1	0/1	0/1
chr2	127070768	CGGAGGCCA	C	624.25	PASS	0/0	0|1	0/0
chr2	127070770	GAGGCC	*,G	501.01	PASS	0/0	1|2	0/0
chr2	127070775	C	CA,*	643.88	PASS	0/1	2|2	0/0

B

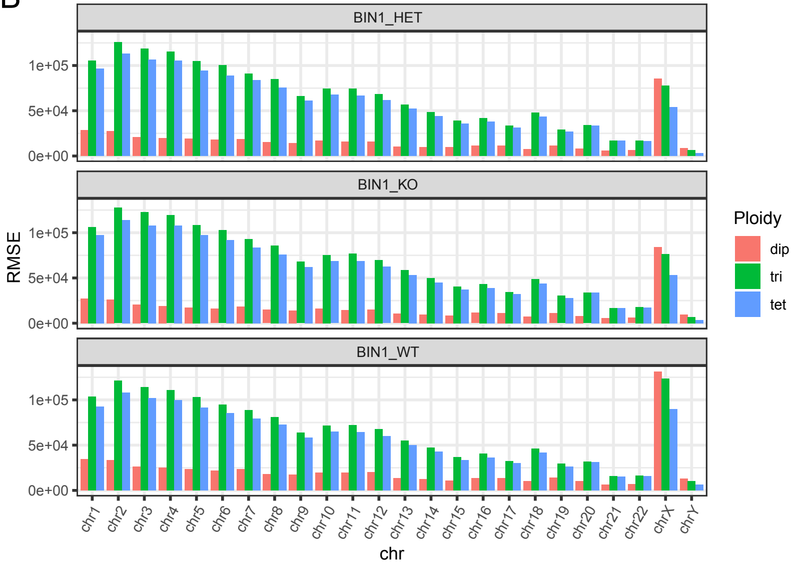

Figure S2

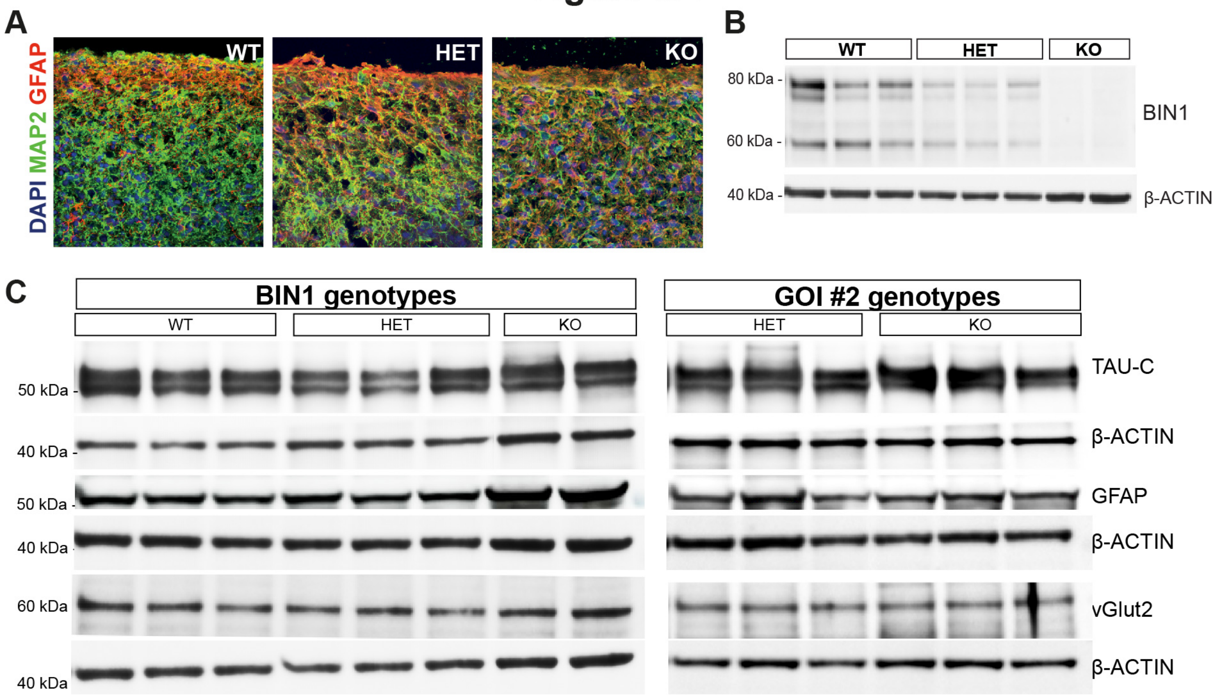

A
DAPI MAP2 GFAP
WT
HET
KO
B
WT
HET
KO
80 kDa
60 kDa
40 kDa
BIN1
β-ACTIN
C
BIN1 genotypes
GOI #2 genotypes
WT
HET
KO
HET
KO
50 kDa
40 kDa
50 kDa
40 kDa
60 kDa
40 kDa
TAU-C
β-ACTIN
GFAP
β-ACTIN
vGlut2
β-ACTIN

Figure S3

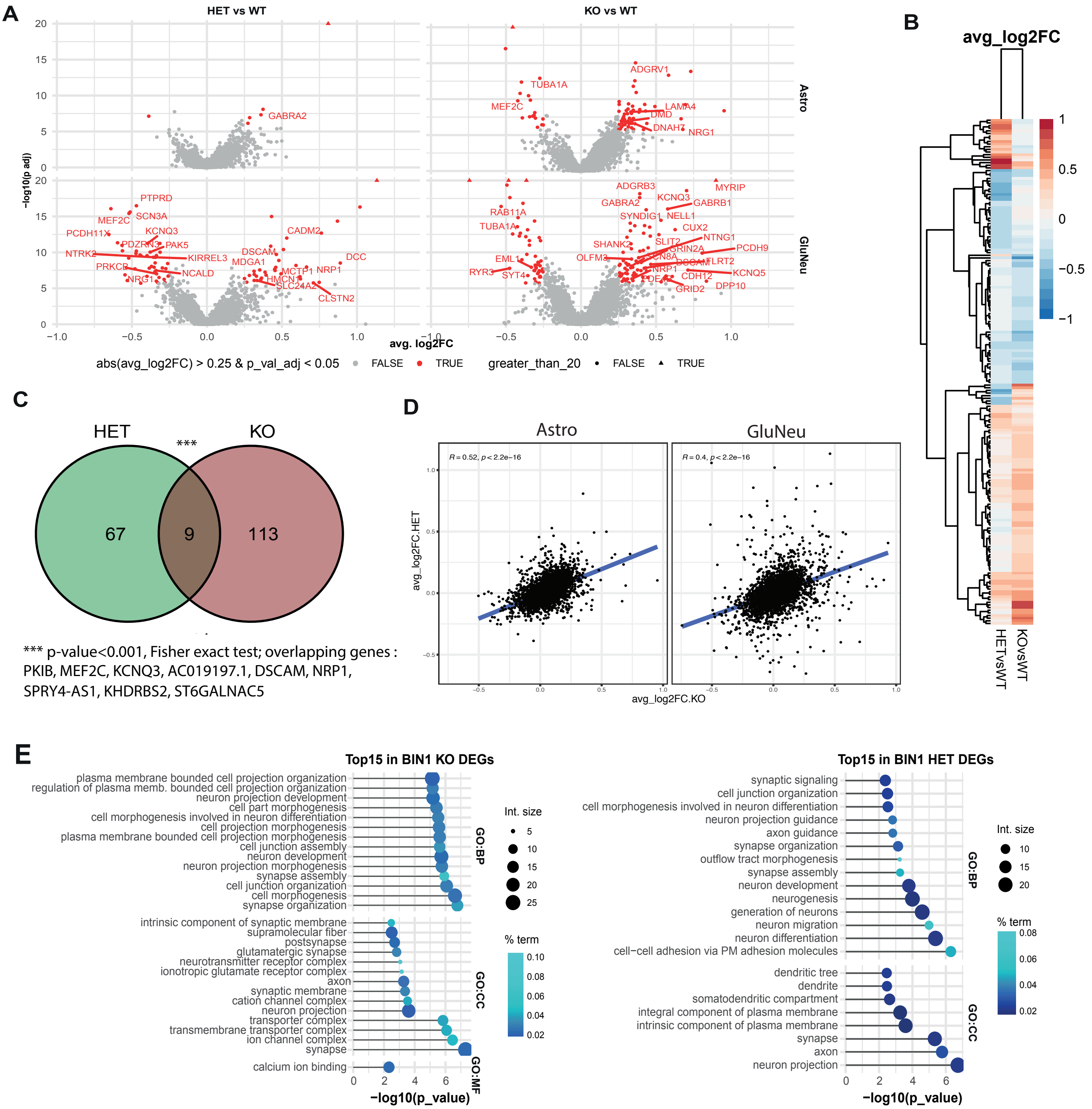
A
HET vs WT
KO vs WT
Astro
GluNeu
−log10(p adj)
avg. log2FC
GABRA2
TUBA1A
ADGRV1
MEF2C
LAMA4
DMD
DNAH7
NRG1
PTPRD
SCN3A
PCDH11X
KCNQ3
PDZRN3
PAK5
NTRK2
KIRREL3
PRKCB
NCALD
NRG1
CADM2
DSCAM
MDGA1
DCC
MCTP1
NRP1
HMCN1
SLC24A2
CLSTN2
ADGRB3
MYRIP
KCNQ3
GABRB1
GABRA2
RAB11A
SYNDIG1
NELL1
TUBA1A
CUX2
NTNG1
SHANK2
SLIT2
PCDH9
GRIN2A
EML1
OLFM3
CN8A
DSCAM
FLRT2
RYR3
SYT4
NRP1
KCNQ5
CDH12
GRID2
DPP10
abs(avg_log2FC) > 0.25 & p_val_adj < 0.05
FALSE
TRUE
greater_than_20
FALSE
TRUE
B
avg_log2FC
HETvsWT
KOvsWT
C
HET
KO
***
67
9
113
*** p-value<0.001, Fisher exact test; overlapping genes :
PKIB, MEF2C, KCNQ3, AC019197.1, DSCAM, NRP1,
SPRY4-AS1, KHDRBS2, ST6GALNAC5
D
Astro
GluNeu
R = 0.52, p < 2.2e−16
R = 0.4, p < 2.2e−16
avg_log2FC.HET
avg_log2FC.KO
E
Top15 in BIN1 KO DEGs
plasma membrane bounded cell projection organization
regulation of plasma memb. bounded cell projection organization
neuron projection development
cell part morphogenesis
cell morphogenesis involved in neuron differentiation
cell projection morphogenesis
plasma membrane bounded cell projection morphogenesis
cell junction assembly
neuron development
neuron projection morphogenesis
synapse assembly
cell junction organization
cell morphogenesis
synapse organization
intrinsic component of synaptic membrane
supramolecular fiber
postsynapse
glutamatergic synapse
neurotransmitter receptor complex
ionotropic glutamate receptor complex
axon
synaptic membrane
cation channel complex
neuron projection
transporter complex
transmembrane transporter complex
ion channel complex
synapse
calcium ion binding
GO:BP
GO:CC
GO:MF
Int. size
% term
−log10(p_value)
Top15 in BIN1 HET DEGs
synaptic signaling
cell junction organization
cell morphogenesis involved in neuron differentiation
neuron projection guidance
axon guidance
synapse organization
outflow tract morphogenesis
synapse assembly
neuron development
neurogenesis
generation of neurons
neuron migration
neuron differentiation
cell−cell adhesion via PM adhesion molecules
dendritic tree
dendrite
somatodendritic compartment
integral component of plasma membrane
intrinsic component of plasma membrane
synapse
axon
neuron projection
GO:BP
GO:CC
Int. size
% term
−log10(p_value)

Figure S4

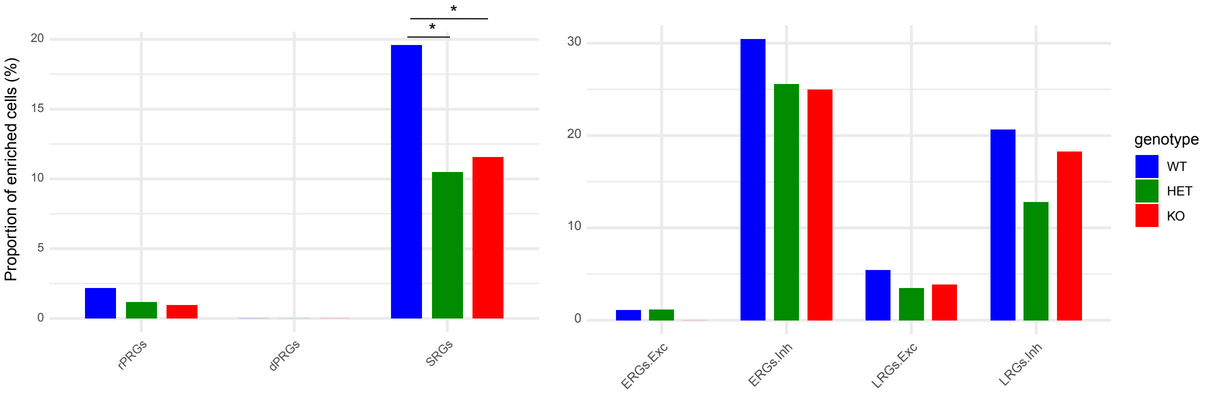

Proportion of enriched cells (%)
20
15
10
5
0
*
*
rPRGs
dPRGs
SRGs
30
20
10
0
ERGs.Exc
ERGs.Inh
LRGs.Exc
LRGs.Inh
genotype
WT
HET
KO

Figure S5

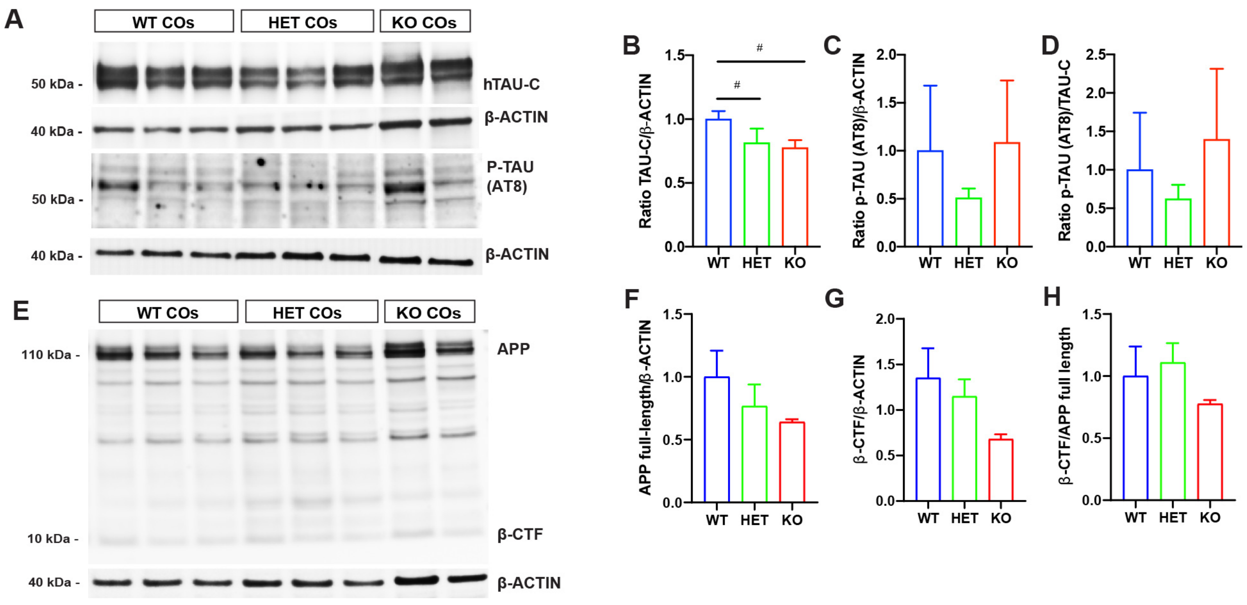

A
WT COs
HET COs
KO COs
50 kDa -
hTAU-C
40 kDa -
β-ACTIN
P-TAU (AT8)
50 kDa -
40 kDa -
β-ACTIN
E
WT COs
HET COs
KO COs
110 kDa -
APP
10 kDa -
β-CTF
40 kDa -
β-ACTIN
B
Ratio TAU-C/β-ACTIN
#
#
WT HET KO
C
Ratio p-TAU (AT8)/β-ACTIN
WT HET KO
D
Ratio p-TAU (AT8)/TAU-C
WT HET KO
F
APP full-length/β-ACTIN
WT HET KO
G
β-CTF/β-ACTIN
WT HET KO
H
β-CTF/APP full length
WT HET KO

Figure S6

A

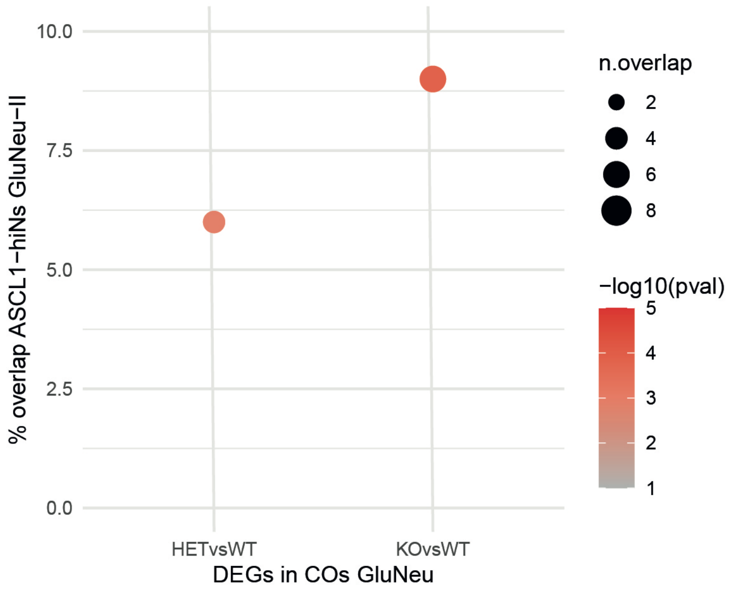

% overlap ASCL1−hiNs GluNeu−II
DEGs in COs GluNeu
HETvsWT
KOvsWT
n.overlap
−log10(pval)

B

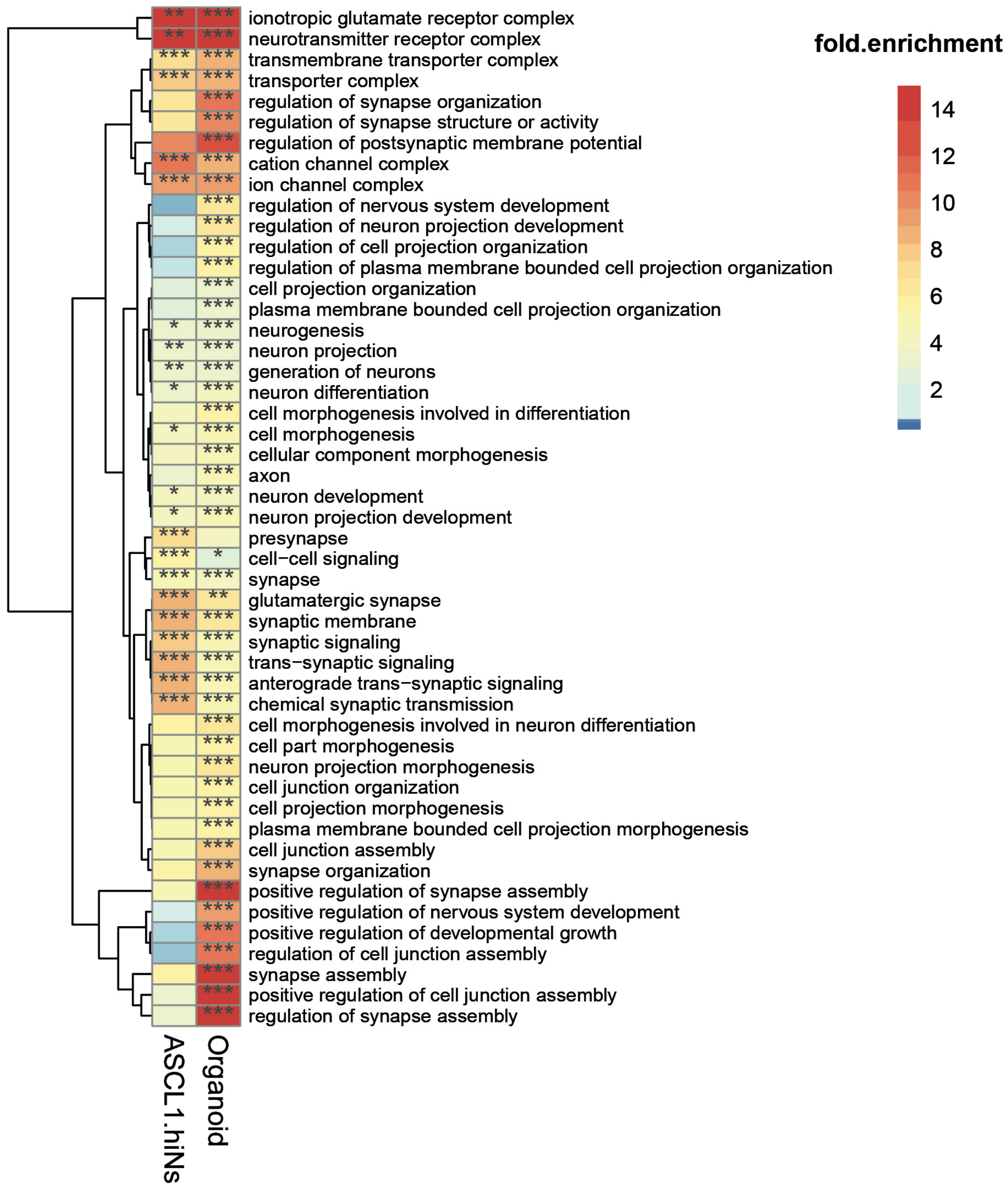

fold.enrichment
ionotropic glutamate receptor complex
neurotransmitter receptor complex
transmembrane transporter complex
transporter complex
regulation of synapse organization
regulation of synapse structure or activity
regulation of postsynaptic membrane potential
cation channel complex
ion channel complex
regulation of nervous system development
regulation of neuron projection development
regulation of cell projection organization
regulation of plasma membrane bounded cell projection organization
cell projection organization
plasma membrane bounded cell projection organization
neurogenesis
neuron projection
generation of neurons
neuron differentiation
cell morphogenesis involved in differentiation
cell morphogenesis
cellular component morphogenesis
axon
neuron development
neuron projection development
presynapse
cell−cell signaling
synapse
glutamatergic synapse
synaptic membrane
synaptic signaling
trans−synaptic signaling
anterograde trans−synaptic signaling
chemical synaptic transmission
cell morphogenesis involved in neuron differentiation
cell part morphogenesis
neuron projection morphogenesis
cell junction organization
cell projection morphogenesis
plasma membrane bounded cell projection morphogenesis
cell junction assembly
synapse organization
positive regulation of synapse assembly
positive regulation of nervous system development
positive regulation of developmental growth
regulation of cell junction assembly
synapse assembly
positive regulation of cell junction assembly
regulation of synapse assembly
ASCL1.hiNs
Organoid

Figure S7

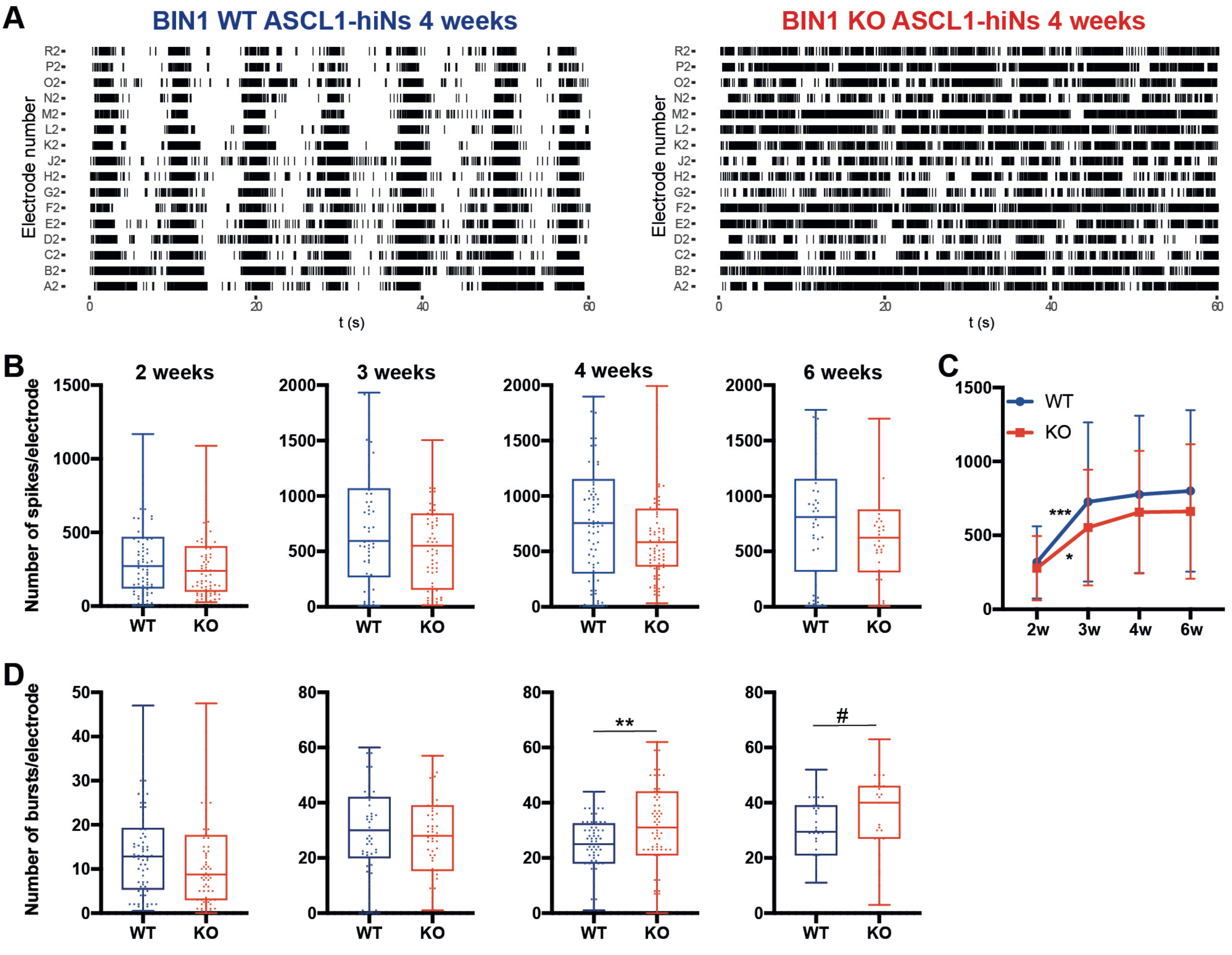

A
BIN1 WT ASCL1-hiNs 4 weeks
BIN1 KO ASCL1-hiNs 4 weeks
Electrode number
R2
P2
O2
N2
M2
L2
K2
J2
H2
G2
F2
E2
D2
C2
B2
A2
t (s)
B
Number of spikes/electrode
2 weeks
3 weeks
4 weeks
6 weeks
WT
KO
C
***
*
2w
3w
4w
6w
D
Number of bursts/electrode
**
#

Figure S8

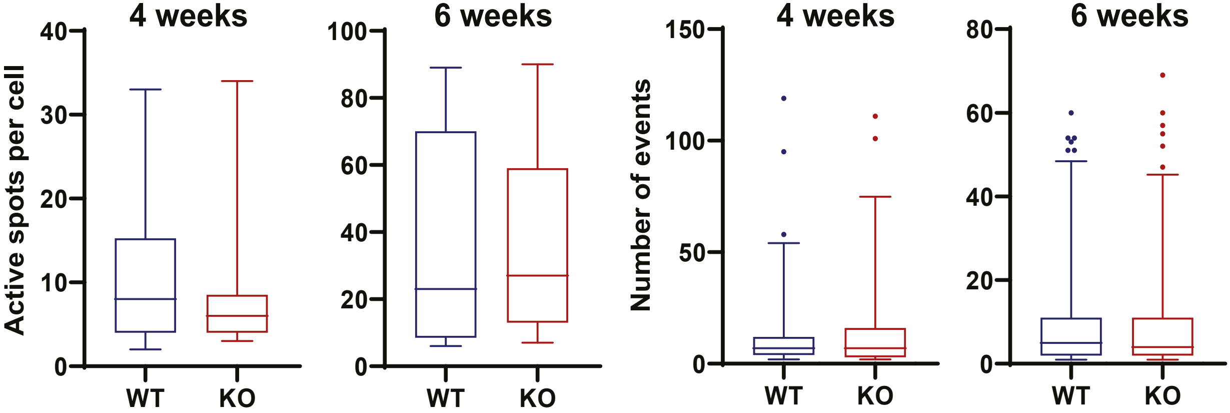

4 weeks
6 weeks
4 weeks
6 weeks
Active spots per cell
Number of events
0
10
20
30
40
0
20
40
60
80
100
0
50
100
150
0
20
40
60
80
WT
KO
WT
KO
WT
KO
WT
KO

Figure S9

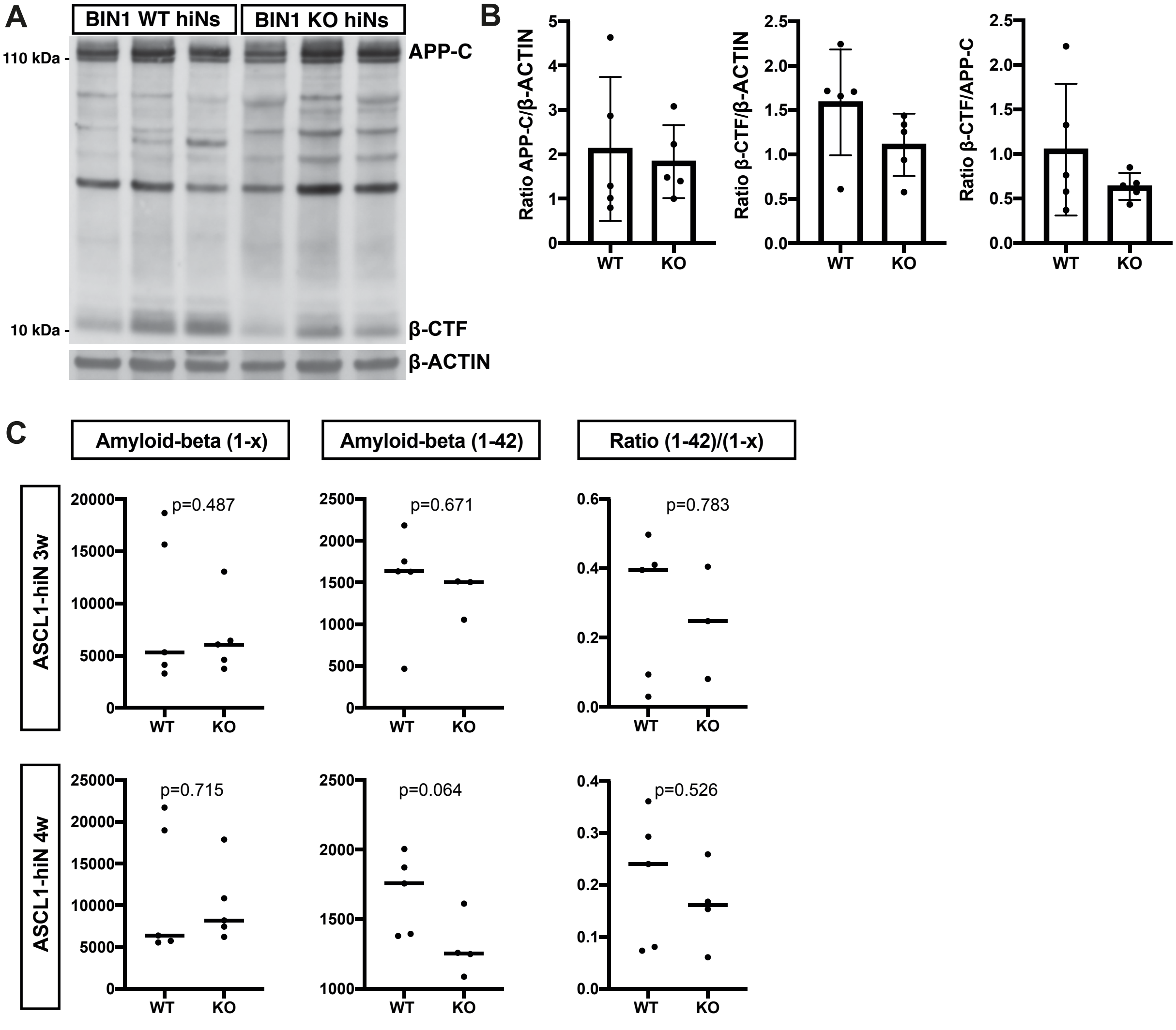

A
BIN1 WT hiNs
BIN1 KO hiNs
110 kDa -
10 kDa -
APP-C
β-CTF
β-ACTIN
B
Ratio APP-C/β-ACTIN
Ratio β-CTF/β-ACTIN
Ratio β-CTF/APP-C
WT
KO
C
Amyloid-beta (1-x)
Amyloid-beta (1-42)
Ratio (1-42)/(1-x)
ASCL1-hiN 3w
ASCL1-hiN 4w
p=0.487
p=0.671
p=0.783
p=0.715
p=0.064
p=0.526

Figure S10

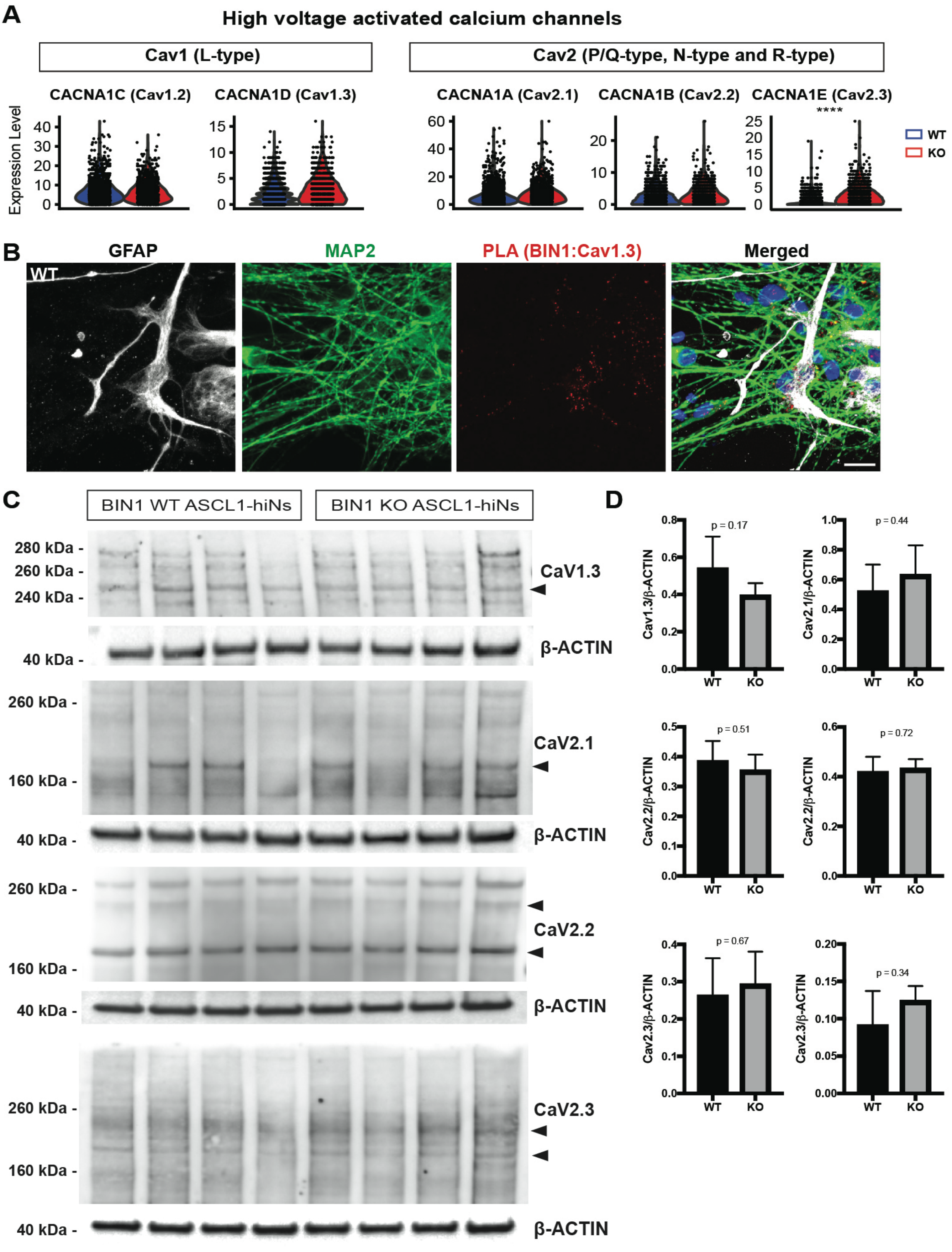
A
High voltage activated calcium channels
Cav1 (L-type)
Cav2 (P/Q-type, N-type and R-type)
CACNA1C (Cav1.2)
CACNA1D (Cav1.3)
CACNA1A (Cav2.1)
CACNA1B (Cav2.2)
CACNA1E (Cav2.3)
****
Expression Level
WT
KO
B
GFAP
MAP2
PLA (BIN1:Cav1.3)
Merged
WT
C
BIN1 WT ASCL1-hiNs
BIN1 KO ASCL1-hiNs
280 kDa -
260 kDa -
240 kDa -
CaV1.3
β-ACTIN
40 kDa -
160 kDa -
CaV2.1
CaV2.2
CaV2.3
D
p = 0.17
p = 0.44
p = 0.51
p = 0.72
p = 0.67
p = 0.34
Cav1.3/β-ACTIN
Cav2.1/β-ACTIN
Cav2.2/β-ACTIN
Cav2.3/β-ACTIN
WT
KO

Figure S11

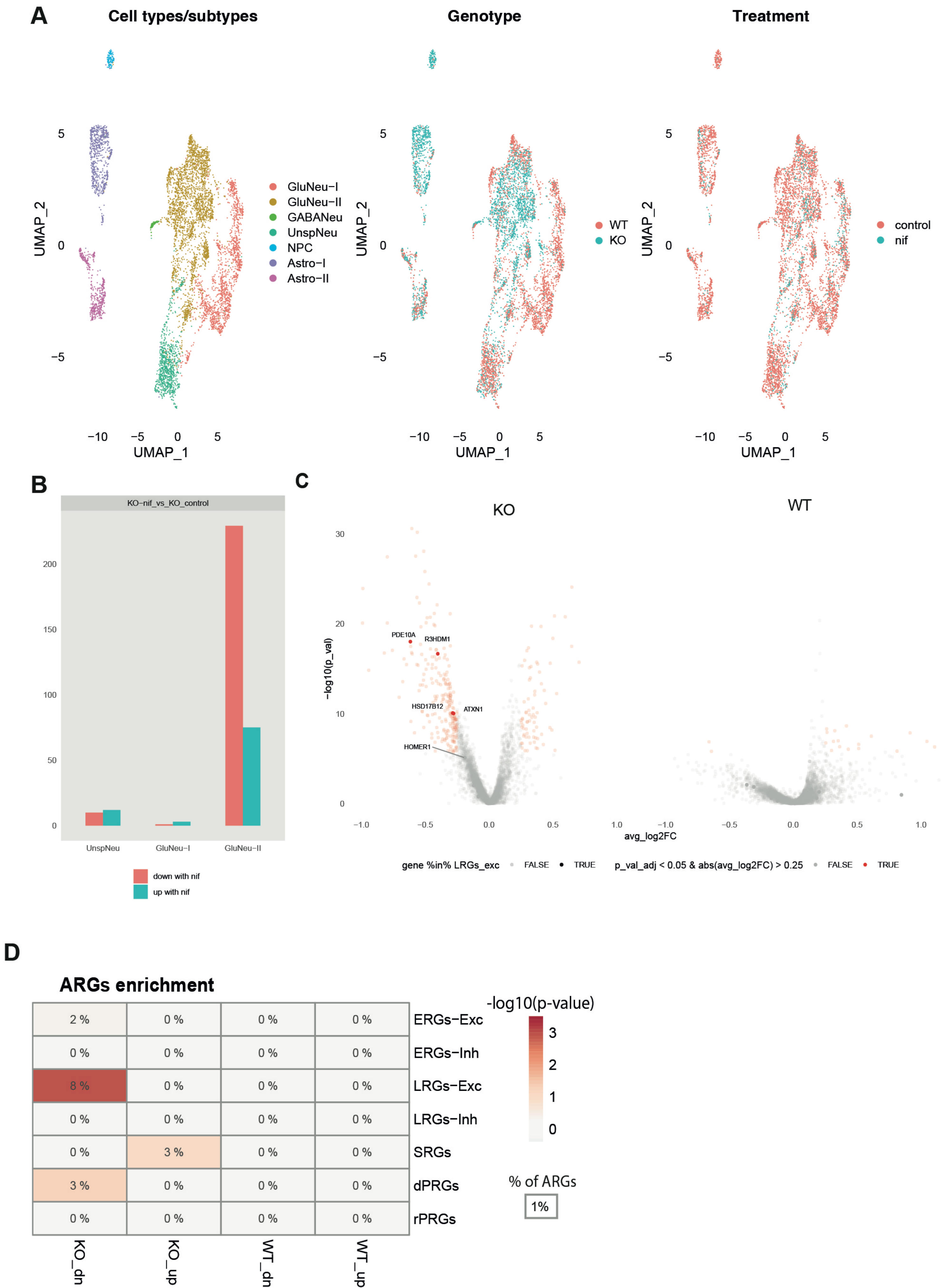
A
Cell types/subtypes
Genotype
Treatment
GluNeu−I
GluNeu−II
GABANeu
UnspNeu
NPC
Astro−I
Astro−II
WT
KO
control
nif
UMAP_1
UMAP_2
B
KO−nif_vs_KO_control
UnspNeu
GluNeu−I
GluNeu−II
down with nif
up with nif
C
KO
WT
−log10(p_val)
avg_log2FC
PDE10A
R3HDM1
HSD17B12
ATXN1
HOMER1
gene %in% LRGs_exc
FALSE
TRUE
p_val_adj < 0.05 & abs(avg_log2FC) > 0.25
D
ARGs enrichment
ERGs−Exc
ERGs−Inh
LRGs−Exc
LRGs−Inh
SRGs
dPRGs
rPRGs
KO_dn
KO_up
WT_dn
WT_up
2 %
8 %
3 %
0 %
-log10(p-value)
% of ARGs
1%

Figure S12

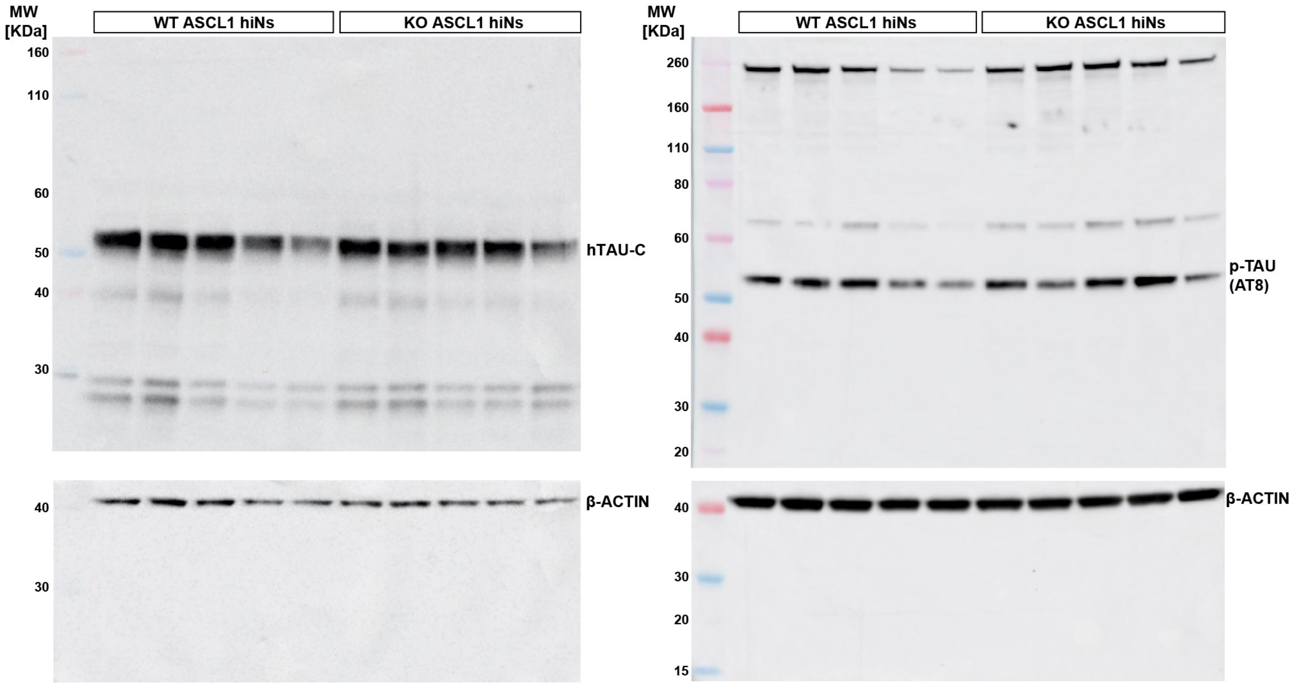

MW [KDa]
WT ASCL1 hiNs
KO ASCL1 hiNs
160
110
60
50
40
30
hTAU-C
40
30
β-ACTIN
MW [KDa]
WT ASCL1 hiNs
KO ASCL1 hiNs
260
160
110
80
60
50
40
30
20
p-TAU (AT8)
40
30
20
15
β-ACTIN

Figure S13

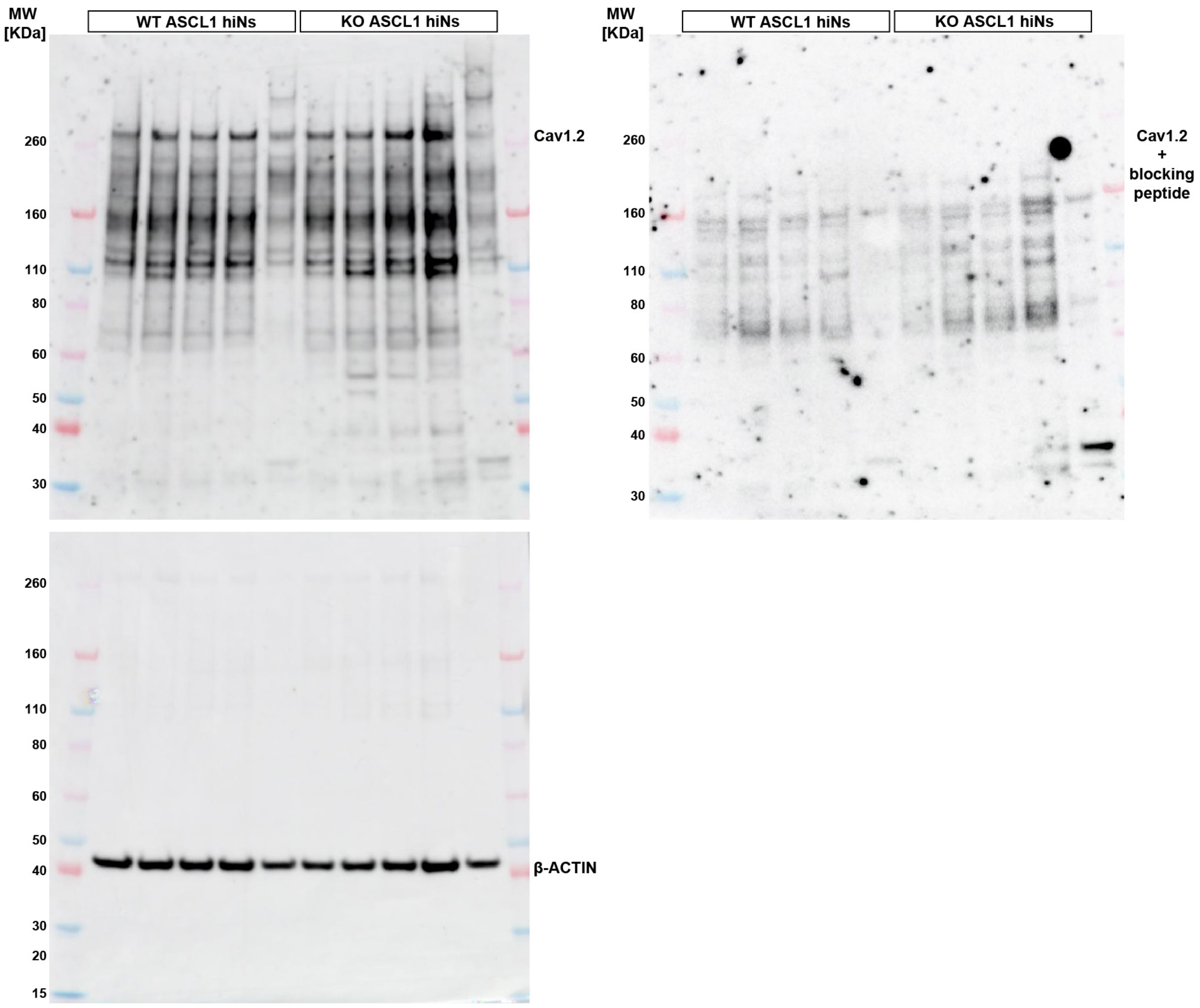

MW
[KDa]
WT ASCL1 hiNs
KO ASCL1 hiNs
260
160
110
80
60
50
40
30
Cav1.2
260
160
110
80
60
50
40
30
20
15
β-ACTIN
MW
[KDa]
WT ASCL1 hiNs
KO ASCL1 hiNs
260
160
110
80
60
50
40
30
Cav1.2
+
blocking
peptide

Figure S14

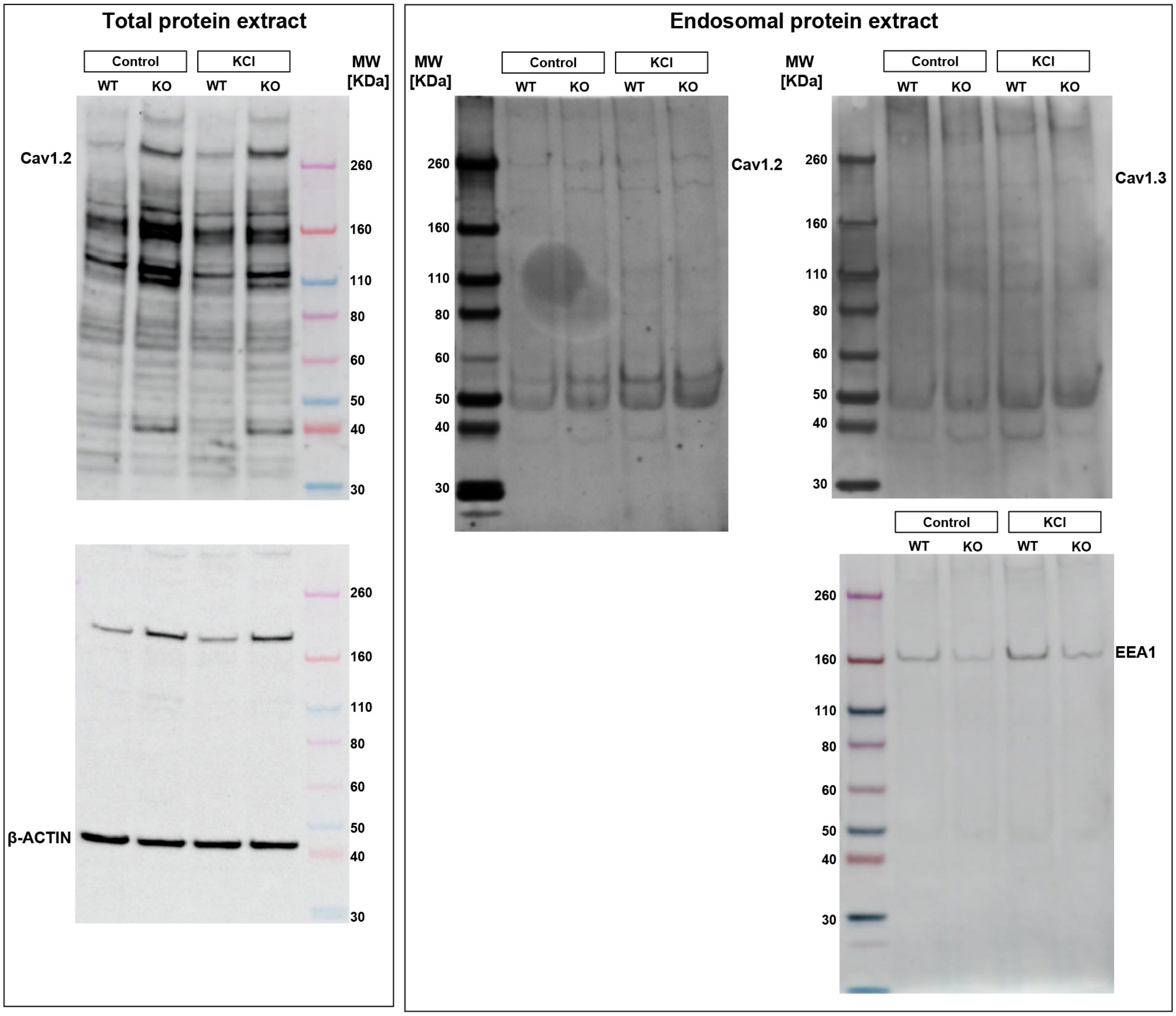
Total protein extract
Control
KCl
WT
KO
WT
KO
MW [KDa]
Cav1.2
260
160
110
80
60
50
40
30
β-ACTIN
Endosomal protein extract
Cav1.3
EEA1

APPENDIX:

Datasheet - Parental hiPSC line ASE-9109 (Applied StemCell Inc)

Project Report I - Cell line Evaluation

Project Report II - Generation of gRNAs

Project Report III - Identification of *BIN1* WT, HET and KO in hiPSC clones

Applied StemCell, Inc.
(866) 497-4180
www.appliedstemcell.com

Datasheet

Induced Pluripotent Stem Cells (Control Line from CD34+ Blood Cells; Male)

Product Information

Catalog Number	**ASE-9109 (Male)**
Description	Applied StemCell, Inc. provides fully characterized control iPSC lines that are derived using a standardized integration-free, episomal reprogramming protocols from CD34+ cord blood cells. The ASE-9109 control line is from a male donor, and has been fully characterized. These include whole genome sequencing, STR tracking, HLA typing, pluripotency marker expression, and the ability to differentiate into the three germ layers as well as terminal stage neural lineage cells. These cell lines have also been used for generating several genome engineered lines. They are ideal as isogenic controls for disease modeling, drug and toxicity screening,
Tissue	CD34+ Cord Blood Cells
Sex	Male
Race	Caucasian
Clinical Information	Normal
Quantity	≥$1.0x10^6$ viable cells/vial
Shipping	Dry ice
Storage and Stability	Store in liquid nitrogen freezer immediately upon receipt. This product is stable for at least 6 months from the date of receiving when stored as directed.
Quality Control	Each lot of human iPS cells has been tested for growth and viability following recovery from cryopreservation. In addition, the iPSCs have also been tested for functional pluripotency (formation of the three germ layers), normal male karyotype (Figure 2), expression of stem cell markers (NANOG, OCT4, TRA-1-81 and TRA-1-60; Figure 1), STR profile (Figure 3); whole genome profiling, ability to differentiate into neuronal lineage cells, and for the absence of mycoplasma and pathogens (CoA available upon request).
Safety Precaution	**PLEASE READ BEFORE HANDLING ANY FROZEN VIALS**. Please wear the appropriate Personal Protection Equipment (lab coat, thermal gloves, safety goggles and a face shield) when handling the cells. Handle the frozen vials with due caution. Please be aware that the following scenario can occur: Liquid nitrogen can leak into the vials when the vials are submerged in liquid nitrogen. Upon thawing, the liquid nitrogen returns to the gas phase, resulting in a dangerous build-up of pressure within the vial. This can result in the vial exploding and expelling not only the vial contents but also the vial cap and plastic fragments of the vial.
Restricted Use	This product is for research use only and not intended for human or animal diagnostic or therapeutic uses.

Applied StemCell, Inc.
521 Cottonwood Dr. #111, Milpitas, CA 95035
Phone: 866-497-4180 (US Toll Free); 408-773-8007 Fax: 408-773-8238
info@appliedstemcell.com www.appliedstemcell.com
Copyright 2017, Applied StemCell, Inc. All rights reserved. This information is subject to change without notice.

Characterization of Control iPSC Lines

Expression of Pluripotency Markers

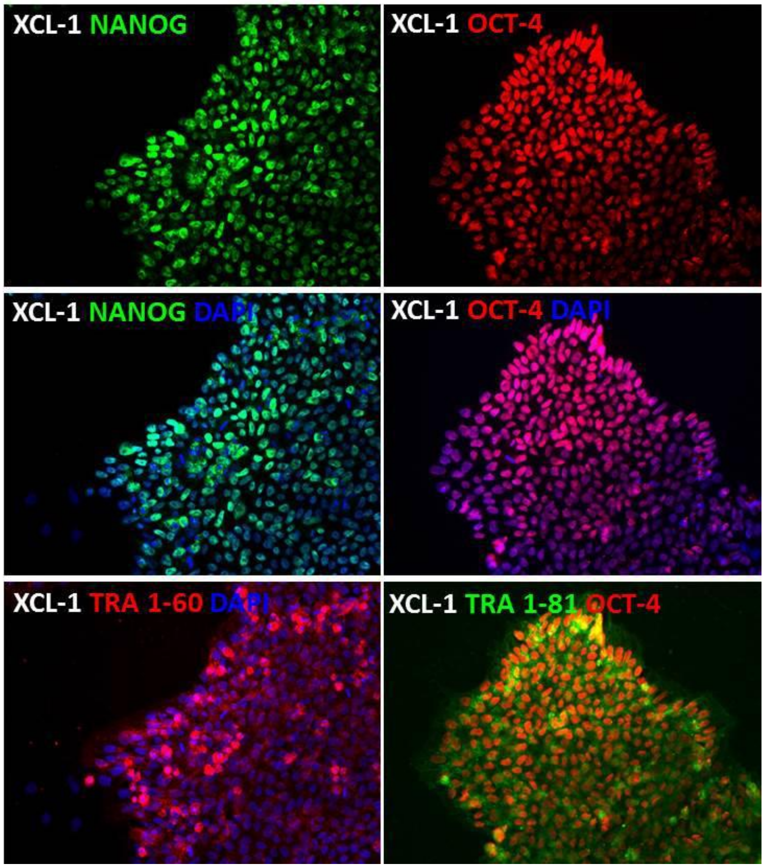

Figure 1. Expression of pluripotency markers in Control iPSC line, ASE-9109. The control iPSC line, derived from male cord blood cells expresses pluripotency markers NANOG, OCT4, TRA-1-60, TRA-1-81 and NANOG indicating pluripotency of the iPSC line after genome editing. Nucleus stained with DAPI (blue).

Karyotype Analysis

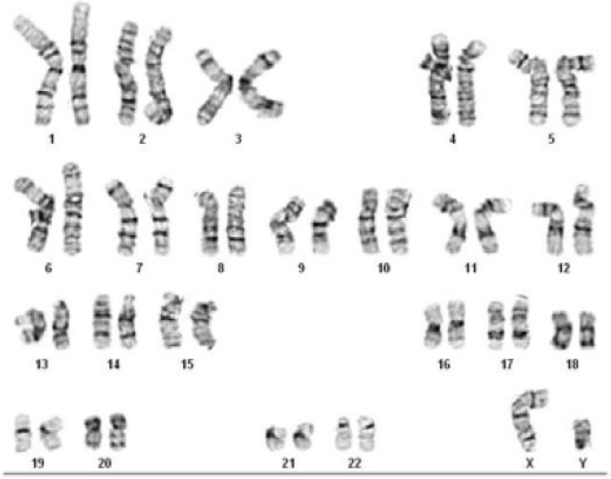

Total counted: 20
Total analyzed: 8
Total karyotyped: 4
Band resolution: 400-450
Normal karyotype. No clonal abnormalities were detected at the stated band level of resolution.

Figure 2. Karyotype Analysis of Control iPSC line, ASE-9109 indicates normal karyotype and no clonal abnormalities at the stated band level of resolution.

STR Profile

STR Profile

Loci	XCL1
AMEL	X,Y
CSF1PO	9,10
D13S317	9,10
D16S539	9,13
D18S51	14,16
D19S433	14
D21S11	29
D2S1338	17,24
D3S1358	14,16
D5S818	10,12
D7S820	8,9
D8S1179	13,15
FGA	21,24
TH01	9,3
TPOX	8,11
VWA	17,18

Figure 3. STR Profiling for Control iPSC line, ASE-9109 derived from male cord blood cells.

Additional Reagents Required but not Provided

- Rock Inhibitor, Stemgent, Cat# ASE-04-0012
- mTeSR™1 Feeder Free Media, STEMCELL Technologies, Cat# 05850
- BD Matrigel™ hESC-qualified Matrix Features, BD Biosciences, Cat# 354277
- DMEM, Life Technologies, Cat# 11995081
- Accutase, Life Technologies, Cat# A1110501
- CryoStem™ Freezing Medium, Stemgent, Cat# 01-0013-50

Equipment Required

Equipment	Specifications
Centrifuge	(e.g. Thermo Centra CL2)
Conical Tubes	Polystyrene Conical Tube 15 mL, Corning Inc., Cat # 430790<br>Polystyrene Conical Tube 50 mL, Corning Inc., Cat# 176740
Cell Counter/Hemocytometer	
Cell Culture Dishes	60mm/15mm, Corning Inc., Cat# 430196

Protocol

All steps must be performed according to standards required for sterile cell culture work.

1. Handling Upon Receiving

Applied StemCell's iPSCs are shipped on dry ice at ambient temperature. Single or multiple vials with cryopreserved cells are packed in a transparent bag, which is buried in dry ice. Upon receiving the product, check the integrity of the packages and the presence of dry ice (contact Applied StemCell, if the integrity of a package has been compromised, e.g. no dry ice in the package).

Cells can be plated immediately after arrival or transferred into liquid nitrogen. Do not remove the vials from dry ice during transportation to storage units. Immediately transfer components (especially the cryopreserved neural stem cells) to storage units, avoiding prolonged exposure to room temperature.

2. Coating Cell Culture Dishes or Flasks with BD Matrigel hESC-qualified Matrix Substrate

Cell culture vessels should be coated one day before or on the day of plating the cells. Please read producer's manual for handling BD Matrigel hESC-qualified Matrix.

Important producer's notes:

It is extremely important that BD Matrigel hESC- qualified Matrix and all culture ware or media coming in contact with Corning Matrigel hESC-qualified Matrix should be pre-chilled/ice-cold since BD Matrigel hESC-qualified Matrix will start to gel above 10°C.

The dilution is calculated for each lot based on the protein concentration. Prepare aliquots according to the dilution factor provided on the Certificate of Analysis (use 1.5-2.0 mL tubes). The volume of the aliquots is typically between 270-350 µL.

2.1 Pre-chill pipette tips and dishes at 4°C.
2.2 Thaw an aliquot (typically between 270-350 µL) of BD Matrigel hESC-qualified Matrix at 4°C (approximately 45-60 minutes).
2.3 Transfer the aliquot on ice into a biological safety cabinet.
2.4 Prepare a 25 mL aliquot of cold DMEM/F12 in 50 mL conical tube and keep on ice.
2.5 Using a p1000 micropipette, transfer 1000 µL of the cold DMEM/F12 from the above tube into the tube with Matrigel, and mix up and down several times. Transfer the Matrigel solution to the 50 mL conical tube containing cold DMEM/F12 and mix several times with a serological pipette (keep on ice).
2.6 Immediately, coat pre-chilled culture dishes with Matrigel/DMEM solution (volume 120-150 µL/cm^2).
2.7 Distribute coating matrix evenly and incubate at room temperature (15-25°C) for at least 1 hour before use.
2.8 Coated dishes can be used immediately or can be stored at 4°C for up to 7 days (aseptic conditions).

Table 1. Recommended volumes of coating reagents for various vessels.

Vessel	Approx. Surface Area	Matrigel
96 well plate	0.33 cm^2/well	50 µL/well
4 or 24 well plate	2 cm^2/well	250 µL/well
35 mm dish	10 cm^2	1.5 mL
60 mm dish	20 cm^2	2.5 mL

3. Recovering iPSC from Frozen Stock (Feeder free, Single Cell Suspension)

3.1 Pre-warm mTeSR1 culture medium to 37°C, and supplement the medium with 10 µM (final concentration of ROCK inhibitor).
3.2 Pre-warm Matrigel coated dishes.
3.3 Pipette out 5 mL of the above medium (mTeSR1 + Rock inhibitor) into a 15 mL conical tube.
3.4 Transfer a vial with frozen iPSC (on dry ice) to the operation side and thaw cells immediately in 37°C degree water bath, until only a small piece of ice is still visible (approximately 1-1.5 minutes).
3.5 Transfer the vial to the biological safety cabinet and transfer thawed iPSC slowly and drop-wise into a 15 mL conical tube containing 5 mL of pre-warmed mTeSR1 + ROCK medium under constant swirling.
3.6 Wash the cryo-vial with additional 1 mL of medium and transfer to the same 15 mL tube.
3.7 Centrifuge cells at 1000 rpm for 5 minutes.
3.8 Aspirate supernatant carefully without disturbing the pelleted cells and re-suspend the pellet carefully but thoroughly (5-6 up-and-down mixes) in 5 mL of fresh mTeSR1 + ROCK medium using 5 mL serological or p1000 micro-pipette with a long tip.
3.9 Count the cells (optional).

3.10 Aspirate Matrigel solution from coated dishes and immediately seed iPSC at 80,000-150,000 cells/cm^2 (do not allow the matrix to dry out). We recommend seeding the entire vial onto one 60 mm dish.

Optional: *Aspirate Matrigel before the cells are thawed and add enough medium to cover the bottom of the culture dish.*

3.11 Distribute cells evenly by performing cross-like moves and place dish(es) in 37°C / 5°CO_2 cell culture incubator.
3.12 Change mTeSR1 medium (without ROCK inhibitor) every day.

4. Enzymatic Passage of Feeder Free Cultured iPSC Using Accutase (Single Cell Suspension)

4.1 Pre-warm plates coated with Matrigel at 37°C.
4.2 Prepare new plate for transfer of clones: Remove the Matrigel solution from the plates and add appropriate volume of pre-warmed mTeSR1 supplemented with 10 µM ROCK inhibitor to each plate (5 mL for 60mm, 10 mL for 100mm plates, etc.).
4.3 Aspirate media from the plate to be passaged. Add pre-warmed Accutase to each plate (2.5 mL for 60mm, 5 mL for 100mm plates), and place the plate in a 37°C incubator. Observe intermittently to determine when cells begin to detach. Do not leave cells in Accutase for longer than 10 minutes.
4.4 When cells are detached, add an equal volume of pre-warmed DMEM to each plate to dilute the Accutase (2.5 mL for 60mm, 5 mL for 100mm plates).
4.5 Pipette the cell mixture in the plates up and down using a p1000 pipette. Wash the bottom of the plate well to ensure detachment of cells. Transfer the cell suspension to a conical tube.
4.6 Centrifuge tubes at 1000 rpm for 5 minutes.
4.7 Aspirate the supernatant and re-suspend cells thoroughly using p1000 pipette in mTeSR1 (4 mL for pellet from 60mm dish, 9 mL for pellet from 100mm dish).
4.8 Centrifuge tube at 1000 rpm for 5 minutes.
4.9 Take 10 µL cell mixture, mix with 10 µL of Trypan blue and use for cell count.
4.10 Plate the cells on the previously prepared Matrigel coated plates with mTeSR1 + ROCK medium at densities ranging from 50,000-100,000 cells/cm^2.
4.11 Incubate at 37°C/ 5°CO_2, and observe attachment within 12 hours. Change media after 24 hours and daily, thereafter.

5. Cryopreservation of iPSCs

5.1 Aspirate media from plates to be harvested. Add pre-warmed Accutase to each plate and place the plate in a 37°C incubator. Observe intermittently to determine when cells begin to detach (approximately 5 minutes). Do not leave cells in Accutase for longer than 10 minutes.
5.2 When cells are detached, add an equal volume of pre-warmed DMEM to each plate to dilute the Accutase, perform cell count and calculate how many vials need to be frozen down (e.g. 2.5 x 10^6 cells per vial).
5.3 Centrifuge the tube at 1000 rpm for 5 minutes.
5.4 Remove supernatant from tube and re-suspend cells in appropriate volume of CryoStem™ freezing media to achieve 2.5 x 106 cells per 0.5 mL.
5.5 Transfer 0.5 mL of the cell suspension in Cryostem™ freezing media to each cryo-vial and place the vials in a freezing container and immediately place the container at -80°C.
5.6 Transfer vials to LN2 within a week's time window.

Cell line Evaluation: ASE-9109(Human)

Customer Information

Project	BIN1 KO in hiPSC line (ASE-9109)
Milestones	ASE-9109, ASC-6012-1a
Job ticket #	C1770
Client name	Marcos Romulado Costa
Client institute	Federal University of Rio Grande do Norte
Report date	2019-02-14

Summary

The goal of this project is to knockout human BIN1 gene in hiPSC cells. To achieve this goal, Applied StemCell (ASC) will use our proprietary CRISPR-Cas9 technology to introduce Cas9/guide RNAs (gRNAs) complexes into the target cells. This report summarizes work for milestone 1, cell line evalutaion, which includes: 1) cell recovery and expansion; 2) mycoplasma test; 3) optimal electroporation conditions to achieve highest level of transfection and minimum cell damage; 4) optimal drug concentrations for transient selection; 5) sequence analysis of the region of interest in target locus.

Results

1. Cell Recovery and Expansion

The recovery and subsequent culture of hiPSC cells was successful.

2. Mycoplasma test (waived)

3. Transfection conditions optimization (waived)

4. Drug kill curve (waived)

Due to the differences in sensitivities to drug, e.g. Puromycin, a range of drug concentrations was tested for conducting effective transient drug selection after transfection. One million electroporated cells were seeded in three wells of a 6-well plate, and drug was added. Best drug selection condition was chosen for future experiments.

5. Sequence analysis of targeting site

iPSC cells were tested for any potential single nucleotide polymorphism (SNP) in the targeted region. Genomic sequence data were aligned with respective sequence acquired from public database. No SNP was observed (**Figure 1**).

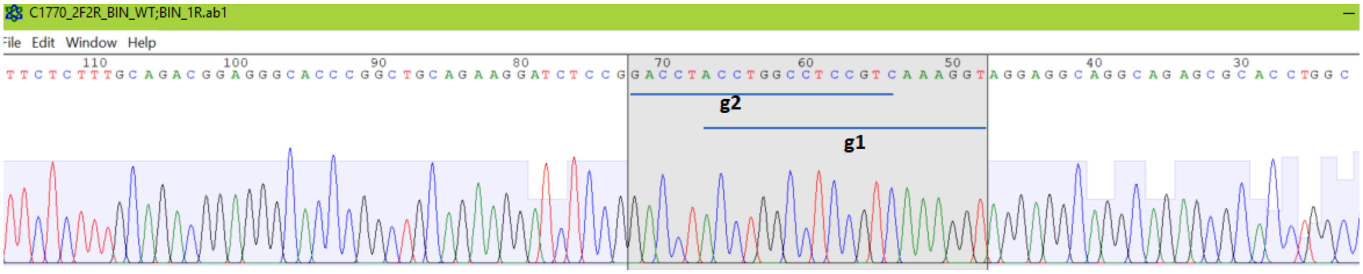

Figure 1. SNP check for gRNA targeted region in host cells.

Generation of gRNAs: BIN1 KO in hiPSC line (ASE-9109) in ASE-9109(Human) Model

Customer Information

Project	BIN1 KO in hiPSC line (ASE-9109)
Milestones	ASC-6012-1b, ASC-6012-1c
Job ticket #	C1770
Client name	Marcos Romulado Costa
Client institute	Federal University of Rio Grande do Norte
Report date	2019-02-14

Summary

The goal of this project is to introduce a knock-out (KO) mutation in the human BIN1 gene in hiPSC cell line. To achieve this goal, Applied StemCell (ASC) will use our proprietary CRISPR-Cas9 technology to introduce Cas9/guide RNA (gRNA) complexes into the target cells. This report summarizes work for milestone 2 and milestone 3, including gRNA construction and reagent validation.

Results

1. gRNA design

The sequences of gRNA candidates are listed in **Table 1** and targeting strategy is illustrated in **Figure 1**. The gRNA candidates were selected based on their predicted activities and off-targeting profiles.

Table 1. Guide RNA candidates and sequences

Name	Sequence (5'→3')
C1770 hBIN1 g1	ACCTTTGACGGAGGCCAGGT
C1770 hBIN1 g2	TGACGGAGGCCAGGTAGGTC

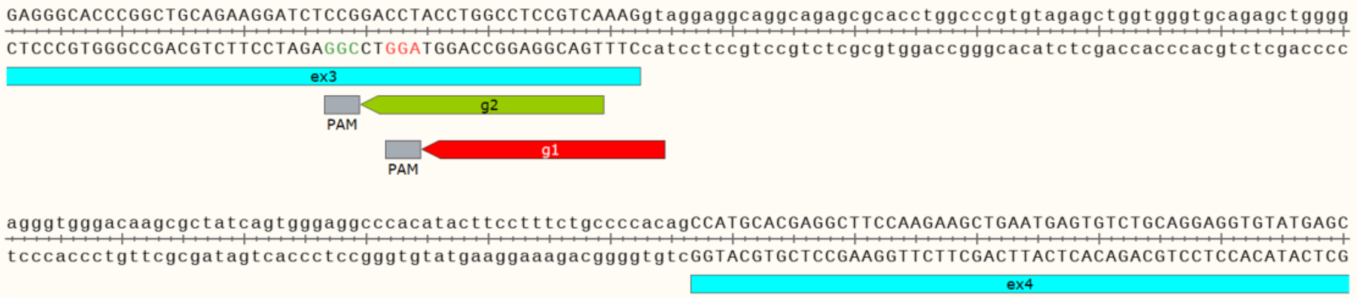

Figure 1. Illustration of gRNA candidates in the targeted gene locus

2. gRNA construction and reagent validation

The above gRNAs were generated and quality control was performed.

QC: Pass.

3. gRNA validation

Each gRNA (g1 and g2) is assessed in host hiPSC cells. Sanger sequencing results showed that both g1 and g2 have good cutting efficiencies (**Figure 2**). g1 was selected to proceed to the next stage.

a) g1

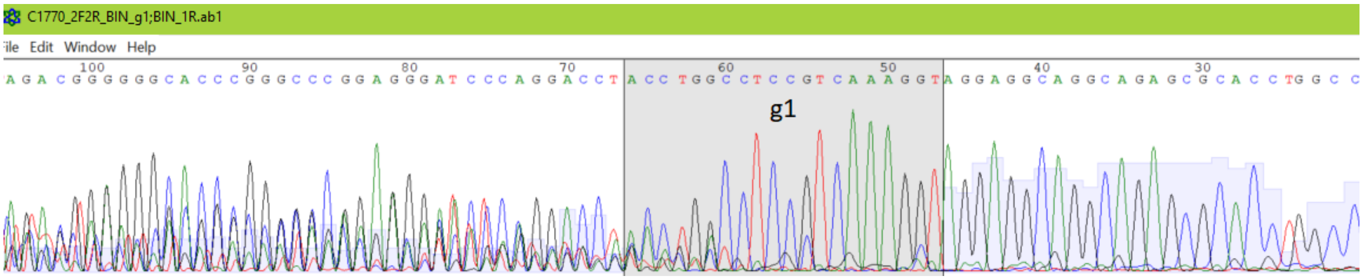

b) g2

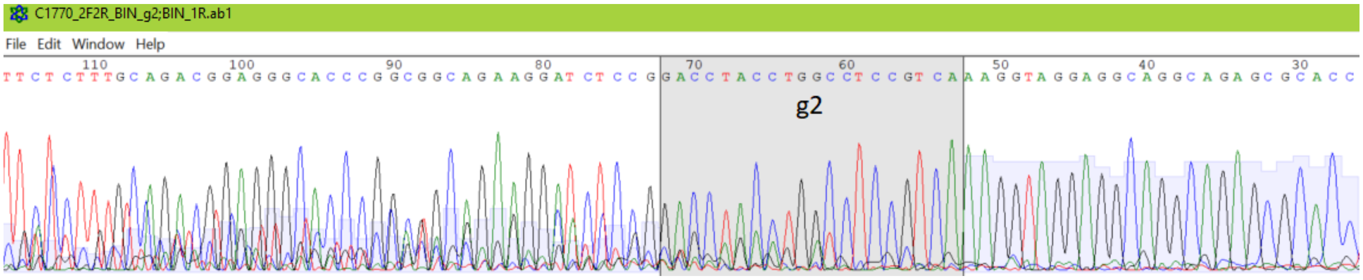

Figure 2. Chromatogram of targeted region in hBIN1 gene after gRNA cutting. Multiple peaks indicate gRNA mediated cleavage in this region; gRNA recognition region highlighted in grey.

Next Step

Applied StemCell will transfect the host iPSC cells with the chosen gRNA followed by single cell cloning in order to isolate clones that have desired KO mutation.

Customer Information

Project	BIN1 KO in hiPSC line (ASE-9109)
Milestones	ASC-6012-1d, ASC-6012-1e, ASC-Custom-4
Job ticket #	C1770
Client name	Marcos Romulado Costa
Client institute	Federal University of Rio Grande do Norte
Report date	2019-05-08

Summary

The goal of this project is to knock out the human BIN1 gene in human iPSC line (ASE-9109). To achieve this goal, Applied StemCell (ASC) used our proprietary CRISPR-Cas9 technology to introduce Cas9/guide RNA (gRNA) complexes into the target cells. Cas9/gRNA mediated indel formation at the targeted region caused by non-homologous end joining (NHEJ) result in frameshift and/or premature stop, thus generating a knockout of the gene of interest. This report summarizes work for milestone 4 and 5, including transfection of targeting vectors and cell confirmation and expansion.

Briefly, based on cutting activities, g1 was chosen to transfect human iPSC cells to generate the knockout clones. After transfecting gRNA and Cas9, single cell-derived clones were screened by sequencing for homozygous and heterozygous clones.

Results

1. Transfection of pooled cells

Host human iPSC cells (ASE-9109) were cultured and electroporated with gRNA g1, based on the conditions as reported (refer to Cell Line Evaluation Report_M1). The electroporation pool was subject to single cell cloning process. In brief, the mixed culture was diluted to less than one cell/200ul culture media and dispersed into each well of a 96-well plate. The cells were allowed to grow for 15 to 20 days, or till a decent number of cells were achieved. Cells derived from such single cell cloning process were subject to genotyping analysis.

2. Screening of positive clones carrying the desired knockout by PCR and sequencing

Genomic DNA from single cell colonies was extracted and PCR was performed to amplify the targeted region. Primer sequences were listed in **Table 1**. Positions of primers are illustrated in **Figure 1**.

Table 1. Primers used for genotyping

Name	Sequence (5' -> 3')	Purpose
C1770 hBIN1 Fwd	gtccctatacaggctcgcac	Amplification of PCR product (719 bp)
C1770 hBIN1 Rev	ttcttggaagcctcgtgcat	Amplification of PCR product (719 bp)
C1770 hBIN1 Rev	ttcttggaagcctcgtgcat	Sequencing PCR product

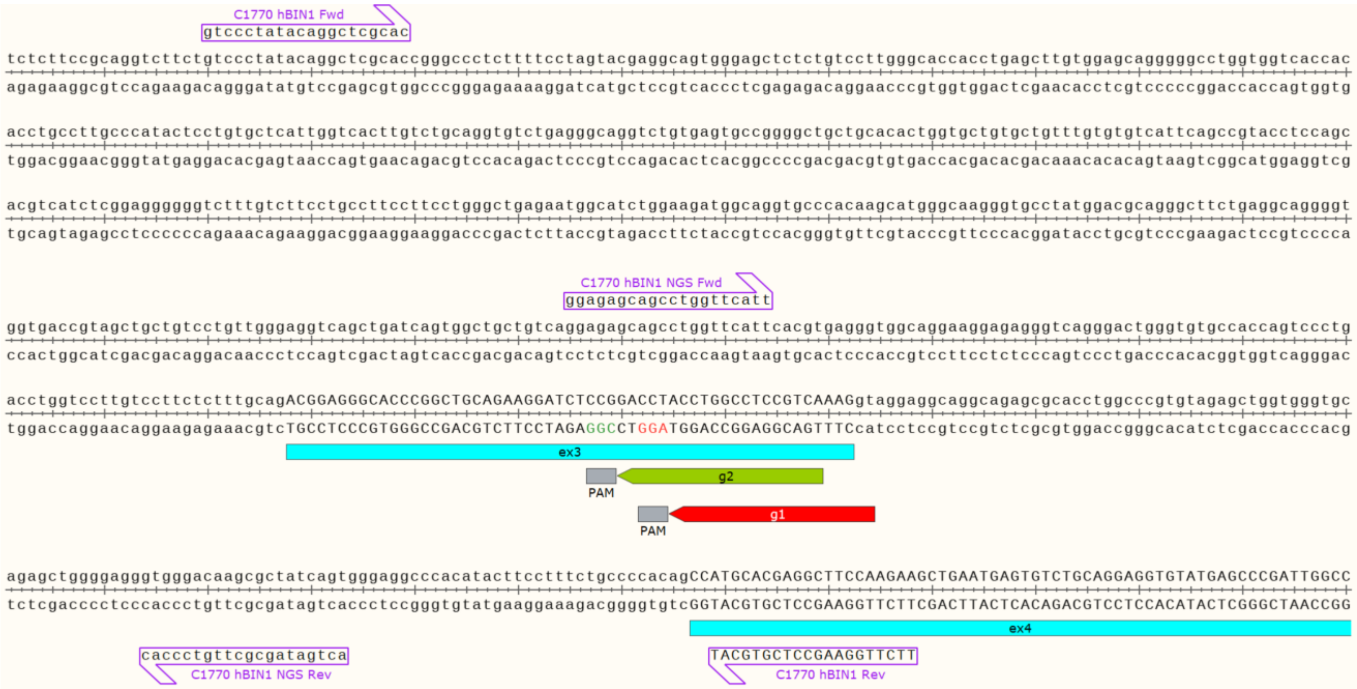

Figure 2. Illustration of the positions of PCR primers and gRNAs in human BIN1 gene

PCR products were further purified and sequenced to identify desired clones. One homozygous clone D6 (-5bp/-8bp functional homozygous), one heterozygous clone C1 (+1bp/WT), and one isogenic WT clone B5 were identified for the desired knockout for hBIN1 gene. (**Figure 3**).

(a) B5 (isogenic WT)

C1770-B5_6874_100	ACCAGTCCCTGACCTGGTCCTTGTCCTTCTCTTTGCAGACGGAGGGCACCCGGCTGCAGA
C1770_ref	ACCAGTCCCTGACCTGGTCCTTGTCCTTCTCTTTGCAGACGGAGGGCACCCGGCTGCAGA
C1770-B5_6874_100	AGGATCTCCGGACCTACCTGGCCTCCGTCAAAGGTAGGAGGCAGGCAGAGCGCACCTGGC
C1770_ref	AGGATCTCCGGACCTACCTGGCCTCCGTCAAAGGTAGGAGGCAGGCAGAGCGCACCTGGC

(b) C1 (heterozygous)

C1770-C1_3862_42.8	ACCAGTCCCTGACCTGGTCCTTGTCCTTCTCTTTGCAGACGGAGGGCACCCGGCTGCAGA
C1770-C1_480_5.3	ACCAGTCCCTGACCTGGTCCTTGTCCTTCTCTTTGCAGACGGAGGGCACCCGGCTGCAGA
C1770-C1_4682_51.9	ACCAGTCCCTGACCTGGTCCTTGTCCTTCTCTTTGCAGACGGAGGGCACCCGGCTGCAGA
C1770_ref	ACCAGTCCCTGACCTGGTCCTTGTCCTTCTCTTTGCAGACGGAGGGCACCCGGCTGCAGA
C1770-C1_3862_42.8	AGGATCTCCGGACCTACCTTGGCCTCCGTCAAAGGTAGGAGGCAGGCAGAGCGCACCTGG
C1770-C1_480_5.3	AGGATCTCCGGACCTACCTTGGCCTCCGTCAAAGGTAGGAGGCAGGCAGAGCGCACCTGG
C1770-C1_4682_51.9	AGGATCTCCGGACCTACC-TGGCCTCCGTCAAAGGTAGGAGGCAGGCAGAGCGCACCTGG
C1770_ref	AGGATCTCCGGACCTACC-TGGCCTCCGTCAAAGGTAGGAGGCAGGCAGAGCGCACCTGG

(c) D6 (homozygous)

C1770-D6_5371_52.2	ACCAGTCCCTGACCTGGTCCTTGTCCTTCTCTTTGCAGACGGAGGGCACCCGGCTGCAGA
C1770_ref	ACCAGTCCCTGACCTGGTCCTTGTCCTTCTCTTTGCAGACGGAGGGCACCCGGCTGCAGA
C1770-D6_583_5.7	ACCAGTCCCTGACCTGGTCCTTGTCCTTCTCTTTGCAGACGGAGGGCACCCGGCTGCAGA
C1770-D6_4338_42.1	ACCAGTCCCTGACCTGGTCCTTGTCCTTCTCTTTGCAGACGGAGGGCACCCGGCTGCAGA
C1770-D6_5371_52.2	AGGATCTCCGGACCTA--------CCGTCAAAGGTAGGAGGCAGGCAGAGCGCACCTGGC
C1770_ref	AGGATCTCCGGACCTAcctggCCTCCGTCAAAGGTAGGAGGCAGGCAGAGCGCACCTGGC
C1770-D6_583_5.7	AGGATCTCCGGACCTA-----CCTCCGTCAAAGGTAGGAGGCAGGCAGAGCGCACCTGGC
C1770-D6_4338_42.1	AGGATCTCCGGACCTA-----CCTCCGTCAAAGGTAGGAGGCAGGCAGAGCGCACCTGGC

Figure 2. Sequencing results of positive clones and isogenic WT clone. a) clone B5 (isogenic WT); b) clone C1 (heterozygous, 1bp insertion on one allele); c) clone D6 (homozygous, 5bp deletion on one allele, 8bp deletion on one allele). Highlighted in yellow: g1 target region in human BIN1.

3. Confirmation and expansion of positive clones carrying the intended knockout

The above clones were subject to expansion. After expansion, a portion of the cells were used for Sanger sequencing to confirm their genotypes. Sequencing results confirmed BIN1 knockout homozygous and heterozygous clones (**Figure 3**). The cells were further cryopreserved in 10% DMSO culture medium in liquid nitrogen, ~ 1X10^6 cells per vial.

(a) B5 (isogenic WT)

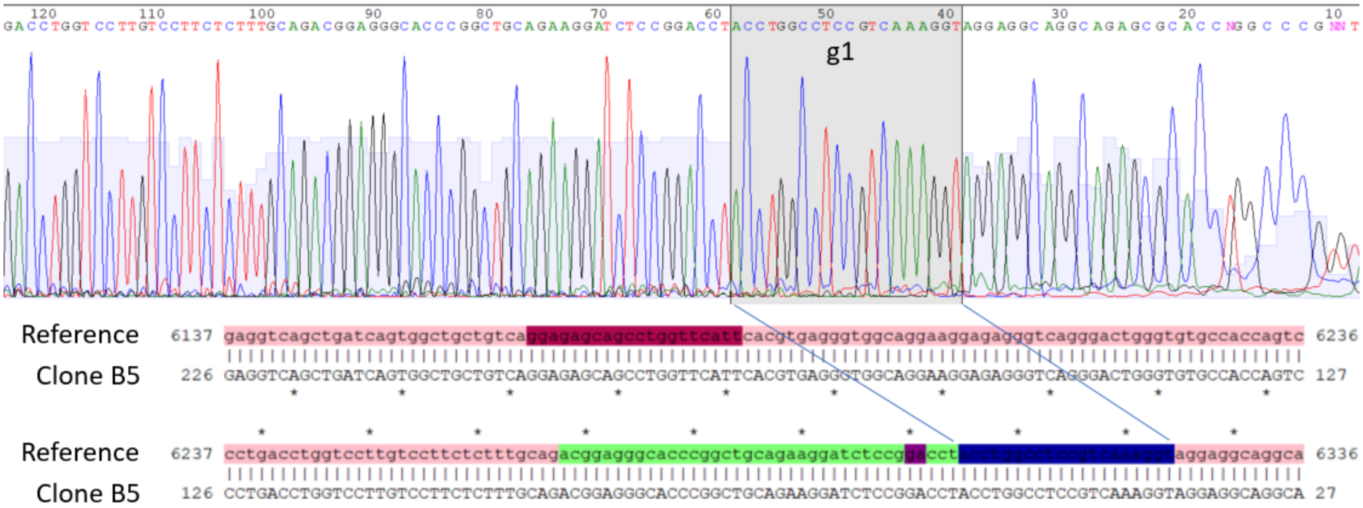

(b) C1 (heterozygous)

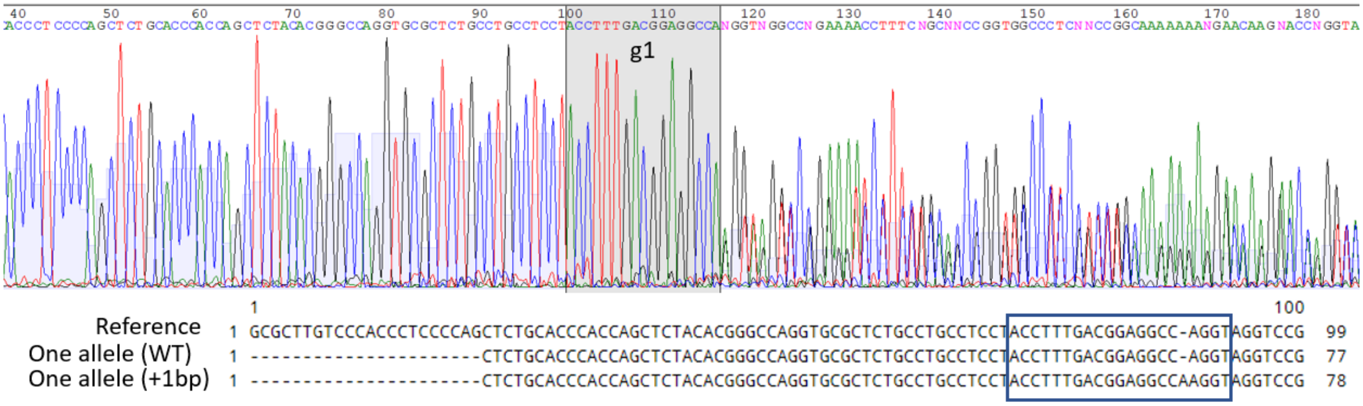

(c) D6 (homozygous)

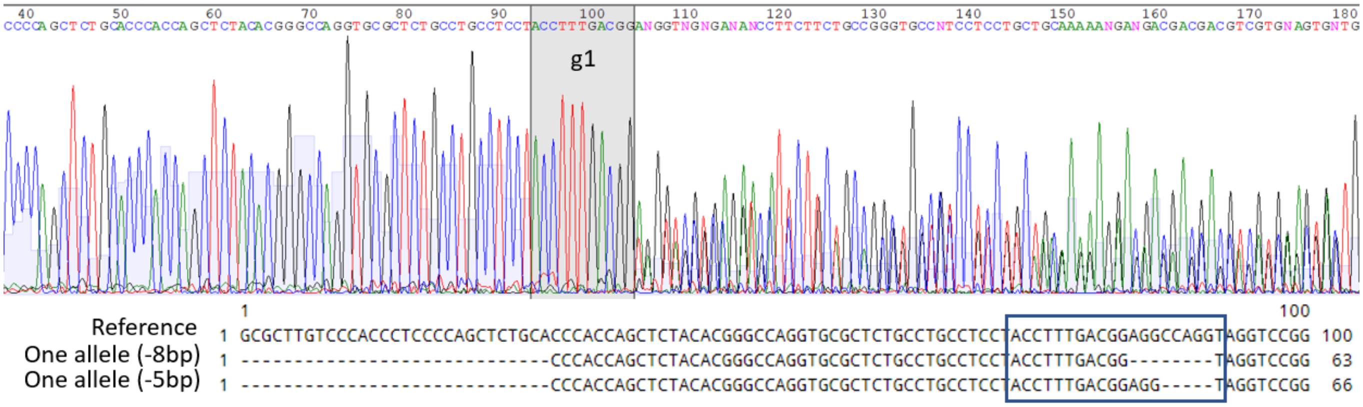

Figure 3. Sequencing result of hBIN1 KO and isogenic WT clones. a) clone B5 (isogenic WT); b) clone C1 (heterozygous, 1bp insertion on one allele); c) clone D6 (homozygous, 5bp deletion on one allele, 8bp deletion on one allele). Highlighted in grey or frame: g1 target region in human BIN1.
